# Supplementary material for: Mechanistic analysis of multiple processes controlling solar-driven H2O2 synthesis using engineered polymeric carbon nitride
Source: Nat Commun. 2021 Jun 17;12:3701. doi: 10.1038/s41467-021-24048-1 (PMC8211848; doi:10.1038/s41467-021-24048-1)
Supplement: Supplementary file 1 — Supplementary Information [file 41467_2021_24048_MOESM1_ESM.pdf]

Supplementary Information

Additional Experimental Details:

Synthesis of mpg-C_3N_4:

mpg-C_3N_4 was synthesized via reported method.[1] In a typical synthesis, 3 gram cyanamide (Sigma-Aldrich) was dissolved in 7.5 g SiO_2 colloid in aqueous suspension (Ludox HS40, Sigma-Aldrich) in a 20 mL glass vial. The transparent liquid mixture was magnetically stirred and heated with oil bath at 70 ^oC overnight. The solid mixture in the glass vial was then transferred into a ceramic crucible with lid. The crucible was heated in a muffle furnace at 823 K for 4 hours under nitrogen flow, and the temperature ramping rate was 2.3 K min^{-1}. The as-obtained powder was pulverized and immersed in 4 M NH_4HF_2 solution for 24 h for template removal, followed by repeated washing with distilled water and ethanol. The powder was finally dried at 323 K under vacuum.

Hydrogen Peroxide Concentration Measurement:

The concentration of the hydrogen peroxide was determined with DPD/POD method reported elsewhere, which is based on the horsedish peroxides (POD)-catalyzed oxidation of N, N-diethyl- p-phenylenediamine (DPD) by hydrogen peroxide.[2] The calibration curve is prepared with a series of H_2O_2 solutions with known concentrations. The concentration of the standard H_2O_2 solution was determined by $KMnO_4$ titration, and $Na_2C_2O_4$ standard solution was employed for the analysis of the concentration of the titrant ($KMnO_4$ solution).

Apparent Quantum Yield (AQY) Measurement:

Reaction mixture was prepared by ultra-sonication dispersion of 10 mg PCN-NaCA-2 in 35 mL glycerol aqueous solution with concentration of 3.5 wt.%. 2 mL of the oxygen saturated reaction mixture was added into a quartz cuvette, which was capped by septum and wrapped by aluminum foil with a 1 cm^2 window. The cuvette was then subject to monochromatic light irradiation for a short time duration, e.g. λ = 380 nm, 420 nm light irradiation for 0.5 min, 450 nm light irradiation for 3 min, 475 nm, 500 nm, and 550 nm light irradiation for 30 min.

The amount of photons (*M*p) absorbed by the photocatalyst was calculated by the equation:

$$Mp = \frac{P_\lambda \times a \times t}{E_\lambda \times N_A} \times (1- 10^{-A})$$

P_λ is the power density on the surface of the cuvette; *a* is the area of the window on the cuvette (0.0001 m^2); *t* is the irradiation time (s); E_λ is the energy per photon with wavelength of λ; N_A is *Avogadro's* constant 6.02×10^{23} mol $^{-1}$; *A* is the absorbance of the cuvette with reaction mixture at λ nm on UV-Vis spectrometer with the water as the background.

The apparent quantum yield (AQY) was calculated by the equation:

$$AQY = \frac{2\times\text{Amount of } H_2O_2}{M_p} \times 100\ \%$$

Photocatalytic Hydrogen Evolution Performance Evaluation:

The photocatalytic H_2 evolution reaction performance was evaluated in a jacked-photoreactor connected to an inner-circulation system under vacuum. H_2 production was quantitatively determined by online gas chromatograph with six-port valve auto-sampling unit. Reaction conditions: 50 mg photocatalyst, 100 mL water, and 10 mL triethanolamine was ultrasonicated and transferred into the jacked-photoreactor. The photoreactor was irradiated by xenon light with a cut-off filter (> 420 nm). The reaction temperature was controlled at 12 ^{o}C by chiller.

Reaction Intermediates Identification:

The aliquot sampled during the photocatalytic reaction was centrifuged, and the supernatant was charged into a glass vial and frozen by liquid-N_2 followed by freeze drying. The concentrated sample was derivatized by silylation. N,O-bis(trimethylsilyl)trifluoroacetamide (BSTFA) containing 1% trimethylchlorosilane (TMCS) and pyridine was added in the glass vial with the concentrated sample and heated at 70 ^{o}C for 3 hours. The mixture was then diluted with dichloromethane for GC-MS analysis.

Characterizations:

FT-IR spectra was collected on Thermo Scientific Nicolet iS 50 with diamond attenuate total reflection (ATR) unit.

The morphology of the catalyst samples were determined by scanning electron microscope (SEM) and high resolution transmission electron microscope (HRTEM) on Hitachi S4300 and JEOL 2100, respectively.

X-ray diffraction (XRD) patterns of the catalysts were obtained on a Rigaku D/MAX 2500 diffractometer with Cu radiation (Cu Kα=0.15406 nm).

X-ray photoelectron spectroscopy (XPS) patterns was recorded on an ESCA laboratory 220i-XL spectrometer with an Al Kα (1486.6 eV) X-ray source and a charge neutralizer; all the binding energy were calibrated to C *1s* peak at 284.6 eV.

BET surface area was measured via nitrogen sorption at 77 K on a surface area analyzer (QuadraSorb SI); the samples were degassed at 200 ^{o}C before nitrogen adsorption.

Contents of nitrogen and carbon in the sample was analyzed with vario El cube (Elementar, Germany).

The UV-Vis absorbance property the samples was recorded on Shimazu UV-2600.

Oxygen temperature programed desorption (O_2-TPD) was measured on Micromeritics AutoChem II 2920. The sample was pretreated in He flow under 300 ^{o}C for 1 hour; and a pulse of 2% O_2 in He was used for absorption of the oxygen molecules on the sample, followed by 1 h He flow with flow rate of 50 mL min^{-1} at 50 ^{o}C for removing the physically adsorbed oxygen molecules. O_2-TPD was measured in the He flow with rate of 50 mL min^{-1}; the

initial temperature was 50 °C, and the ramp rate was 15 °C min^{-1}. Desorbed oxygen was monitored by thermal conductivity detector (TCD).

Thermogravimetric-Infrared-Gas Chromatography/Mass Spectrometry (TG-IR-GC/MS) analysis was realized on PerkinElmer hyphenated multi-detector instrument combining TGA 8000 thermal gravimetric analyzer, FRONTIER infrared spectrometer, and Clarus SQ 8 Gas Chromatograph/Mass Spectrometer. GC was installed with Elite-5MS capillary column (30 m, 0.25 mm ID, 0.25 μm df). Temperature program: initial temperature, 60 °C and hold for 2 min; ramp rate 10 °C min^{-1}; final temperature, 280 °C and hold for 3 min.

Reaction intermediates and products were analyzed by GCMS-QP2020 NX gas chromatograph-mass spectrometer (GC-MS). Gas chromatograph was installed with SH-Rxi™-5Sil MS capillary column (30 m, 0.25 mm ID, 0.25 μm df). Temperature program: initial temperature: 50 °C and hold for 2 min; ramp rate, 15 °C min^{-1}; final temperature, 280 °C and hold for 2 min.

SPV and TPV measurement:

The SPV spectra measurement was conducted based on the lock-in amplifier.[3,4] The measurement system consists of a 500 W xenon lamp with monochromator (SBP500, Zolix) as light sourve, a lock-in amplifier (SR830, Stanford Research Systems, Inc.) with a light chopper (SR540, Stanford Research Systems, Inc.), and a sample chamber. The monochromatic light was chopped with a frequency of 23 Hz. The monochromator and the lock-in amplifier were controlled by a computer. The input resistance of the lock-in amplifier is 10 MΩ. SPV spectra were carried out by scanning from low photon energy to high.

The TPV measurement system consists of a Nd:YAG laser source (Polaris II, New Wave Research, Inc.) providing 355 nm laser pulse radiation, a 500 MHz digital phosphor oscilloscope (TDS 5054, Tektronix) with a preamplifier, and a sample chamber. The oscilloscope was triggered by a synchronous signal provided by a photomultiplier. The input resistance of the preamplifier is 100 MΩ.

Mott–Schottky plots measurement

The sample film was applied on the FTO electrode by spin coating. The slurry was prepared by mixing 20 mg catalyst, 2.7 mL of isopropyl alcohol, 0.9 mL H_2O, and 40 μL Nafion solution (5%, Sigma-Aldrich) under ultrasonication. The impedance-potential values for Mott–Schottky plots were measured in nitrogen-saturated 0.2 M Na_2SO_4 with Ag/AgCl as the reference electrode and Pt as the counter electrode. The data was collected in the voltage range of –0.5 V to +1.3 V at 1000 Hz frequency.

Computational methods:

All spin-unrestricted calculations were performed within the DFT framework based on $DMOL^3$ code.[5,6] The generalized gradient approximation (GGA) with the Perdew-Burke-Ernzerhof (PBE) functional was used to describe exchange and correlation effects.[7] The All Electron Relativistic core treatment method was selected for relativistic effects, which explicitly includes all electrons and introduces some relativistic effects into the core.[8] The double numerical atomic orbital augmented by a polarization function was employed as the basis set. A smearing of 0.005 Ha to the orbital occupation was applied to achieve accurate electronic convergence. The convergence tolerances of energy, maximum force and displacement were 1.0×10^{-5} Ha, 0.002 Ha/Å, and 0.005 Å, respectively. A

conductor-like screening model (COSMO) was used to simulate a H_2O solvent environment throughout the whole process.[9] The orthorhombic supercells for PCN and PCN-NaCA were constructed in sizes of 6.95 × 23 × 20 and 13.99 × 23 × 20 $Å^3$, respectively. Such large cells were required to minimize the interaction between tri-s-triazine units in adjacent cells. The convergence test results for the lattice of PCN-NaCA is shown in Table S3. To describe the van der Waals (vdW) interaction, the DFT + vdW approach within Grimme scheme was adopted.[10]

The adsorption energies (E_{ad}) of adsorbates on PCN and PCN-NaCA were calculated as:

$$E_{ad} = E_{ads} + E_{PCN(PCN\text{-}NaCA)} - E_{ads/PCN(PCN\text{-}NaCA)}$$

where $E_{ads/PCN(PCN\text{-}NaCA)}$ is the total energy of the system with adsorbates on the surface, E_{ads} and $E_{PCN\ (PCN\text{-}NaCA)}$ are the energy of an isolated adsorbate molecule and PCN (or PCN-NaCA), respectively. Based on these definitions, a positive E_{ad} value corresponds to an exothermic stable adsorption process.[11,12]

The free energies of elemental reaction steps were calculated by the computational hydrogen electrode (CHE) model developed by Nørskov *et al.*[13,14] The CHE model defines that the chemical potential of one proton/electron in solution is equal to one half of the chemical potential of one hydrogen molecule in gas-phase H_2.

The free energy (ΔG) for elemental reaction step were calculated as:

$$\Delta G = \Delta E + \Delta E_{ZPE} - T\Delta S + \Delta G_{U} + \Delta G_{pH}$$

where ΔE is the difference between the total energy, ΔE_{ZPE} and ΔS are the differences in the zero-point energy and the change of entropy, T is the temperature (T = 298.15 K in this work), ΔG_U and ΔG_{pH} are the contributions from the electrode potential (U) and pH value, respectively.

The effect of electrode potential U (ΔG_U) is determined as:

$$\Delta G_U = -neU$$

where n is the number of electrons transferred in the elemental step, and U is the electrode potential.

The effect of pH (ΔG_{pH}) is obtained as:

$$\Delta G_{pH} = pH \times k_B T \ln 10$$

where pH equals to 9 (alkaline condition in the experimental measurements), and k_B is Boltzmann constant.

The gas-phase H_2O and H_2 were used as reference states. Due to the fact that the high-spin ground state of O_2 molecule is notoriously poorly described in DFT calculations, the free energy of O_2(g) ($G_{O_2(g)}$) was derived as:

$$G_{O_2(g)} = 2\ G_{H_2O(l)} - 2\ G_{H_2(g)} + 4.92\ \text{eV}$$

In this work, both $2e^-$ and $4e^-$ ORR pathways were considered. The $2e^-$ ORR process in alkaline media is proposed as following chemical equations:

$O_2(g) + * \rightarrow O_2*$ (1)

$O_2^* + H_2O + e^- \rightarrow OOH^* + OH^-$ (2)

$OOH^* + H_2O + e^- \rightarrow H_2O_2 + OH^- + *$ (3)

The 4e⁻ pathway of the ORR process in alkaline media is proposed as following chemical equations:

$O_2(g) + * \rightarrow O_2^*$ (1)

$O_2^* + H_2O + e^- \rightarrow OOH^* + OH^-$ (2)

$OOH^* + e^- \rightarrow O^* + OH^-$ (4)

$O^* + H_2O + e^- \rightarrow OH^* + OH^-$ (5)

$OH^* + e^- \rightarrow OH^- + *$ (6)

where * denotes the surface-active site.

It was found that on an electrically neutral cell (of both PCN and PCN-NaCA), ORR process is unlikely to happen, because the initial step of ORR, i.e., O_2 adsorption, is energetically unfavorable (Figure S33). When the cell is negatively charged with one additional electron, ORR on PCN and PCN-NaCA can proceed with surmountable barriers, which is consistent with findings by Qiao's group.[15] To check the possible interaction between the positive charge background and the COSMO model, we examined the adsorption energy of OOH on PCN-NaCA with a "cell-extrapolation" method using increasingly larger cells;[16] there is no obvious change in the adsorption energy of OOH on PCN-NaCA observed with the increase of the cell dimensions (Table S4). The substrate, negatively charged by one additional electron, is thus employed in all the calculations in this work, unless stated otherwise.

Supplemental Figures, Tables, and Notes

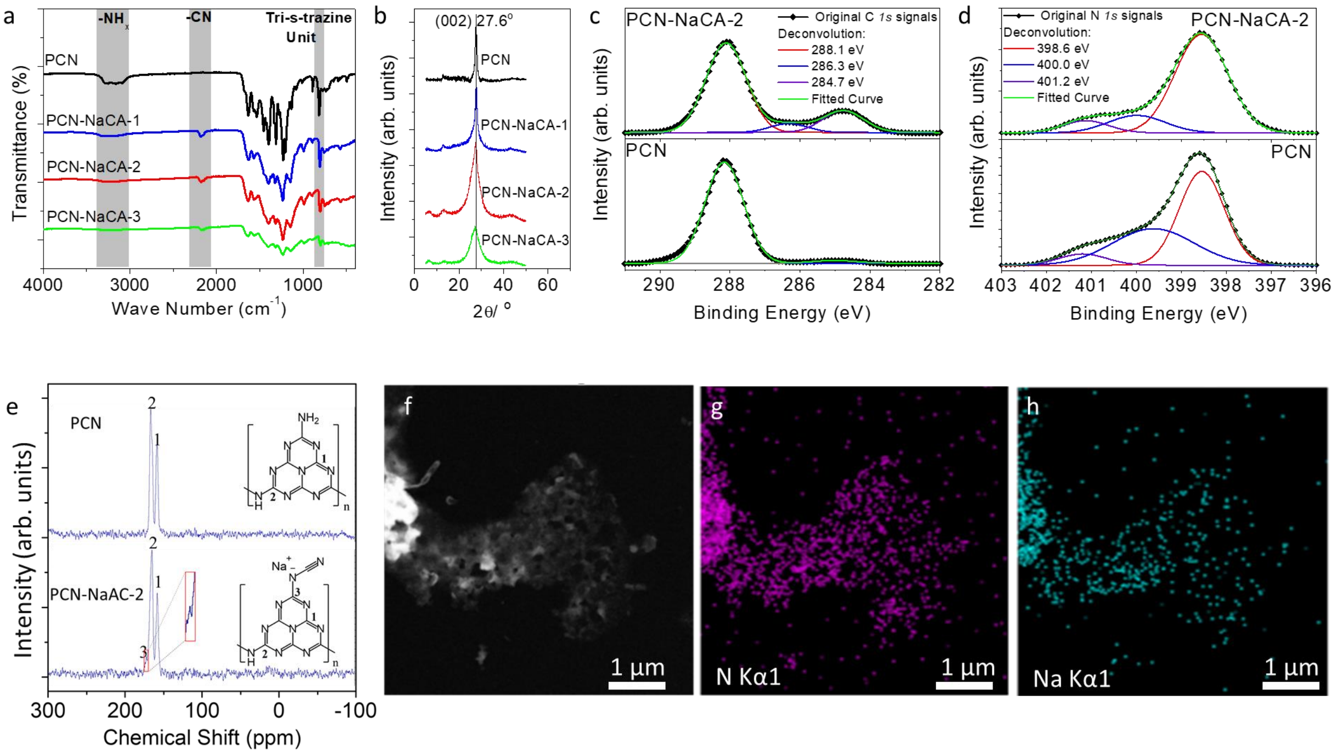

Figure S1. Characterization of the photocatalysts. a FT-IR spectra of the photocatalysts. **b** X-ray diffraction patterns of the samples. **c, d** C_{1s} and N_{1s} XPS profiles of PCN and PCN-NaCA-2 and their respective deconvoluted peaks. **e** ^{13}C-NMR spectra of PCN and PCN-NaCA-2. STEM image (**f**), EDS mapping of nitrogen (**g**) and sodium (**h**) of PCN-NaCA-2.

Interpretation of the characterizations:

FT-IR spectra: The absorption peak at 808 cm^{-1} is due to the out-of-plane bending of the tri-s-triazine unit. For PCN, strong and broad absorption peak at 3000 cm^{-1} to 3500 cm^{-1} from the N-H stretching vibration is observed, stating the presence of rich amino groups.[17] With the increase of the salt/PCN ratio (from PCN-NaCA-1 to PCN-NaCA-3), the asymmetric vibration peak of cyano group at 2175 cm^{-1} grows, and the N-H vibration peak decreases simultaneously, demonstrating the conversion of the amino group to the cyanaminate moiety.

XRD patterns: The PCN presents a strong and sharp X-ray diffraction peak at 27.6 °, demonstrating the layer stacking structure on (002) direction with interlayer spacing of 0.33 nm. All PCN-NaCA-n samples present the same diffraction angle, i.e., the introduction of sodium cyanaminate moiety does not alter the interlayer spacing of the stackings. However, further polymerization reaction in molten salt changes the intensity and width of the diffraction peak. As compared to PCN, PCN-NaCA-2 shows much stronger and wider diffraction peak. This states that further polyerization reaction in the molten salt produces new carbon nitride layer stackings with much thinner thickness than that of PCN.

XPS profiles: For the sample PCN, C *1s* peaks can be deconvoluted into three peaks at 288.1 eV, 286.3 eV, and

284.7 eV, which are respectively assigned to the N−C=N in the heptazine unit, the carbon atoms connected to $-NH_{2/1}$ or cyano groups, and the adventitious carbon (C−C or C=C).[18] In C_{1s} profile of PCN-NaCA-2, the percentage of the deconvoluted peaks at 286.3 eV increased. Based on the analysis on the FT-IR and ^{13}C spectra, this intensified peak is repeatedly stating the formation of the cyanaminate moiety on the edge of the carbon nitride framework.

The N *1s* peak at 398.6 eV is assigned to nitrogen functional forms of pyridinic N and cyano group; and the peaks around 400 eV is assigned to amine group; the binding energy peak at 401 eV is usually attributed to the graphitic N.[19,20,21] As compared with PCN, PCN-NaCA-2 shows increased ratio of the 398.6 eV component, and decreased ratio of 400 eV component. This could be attributed to the conversion of the amino group to the cyanaminate moiety, which is consistent to the FTIR spectra.

^{13}C NMR spectra: In the ^{13}C NMR spectra of PCN and PCN-NaCA-2, the peaks at 157 ppm and 164 ppm are assigned to C(3N) and C(2N, NH_x), respectively. For the sample PCN-NaCA-2, there is an additional peak appearing at 171 ppm is assigned to the carbon connecting with the sodium cyanaminate moiety.[22]

STEM-EDS: Uniform distribution of sodium in the matrix is observed.

Elemental Analysis: The C/N ratio of the framework is, respectively, 0.685 and 0.719 for PCN and PCN-NaCA-2.

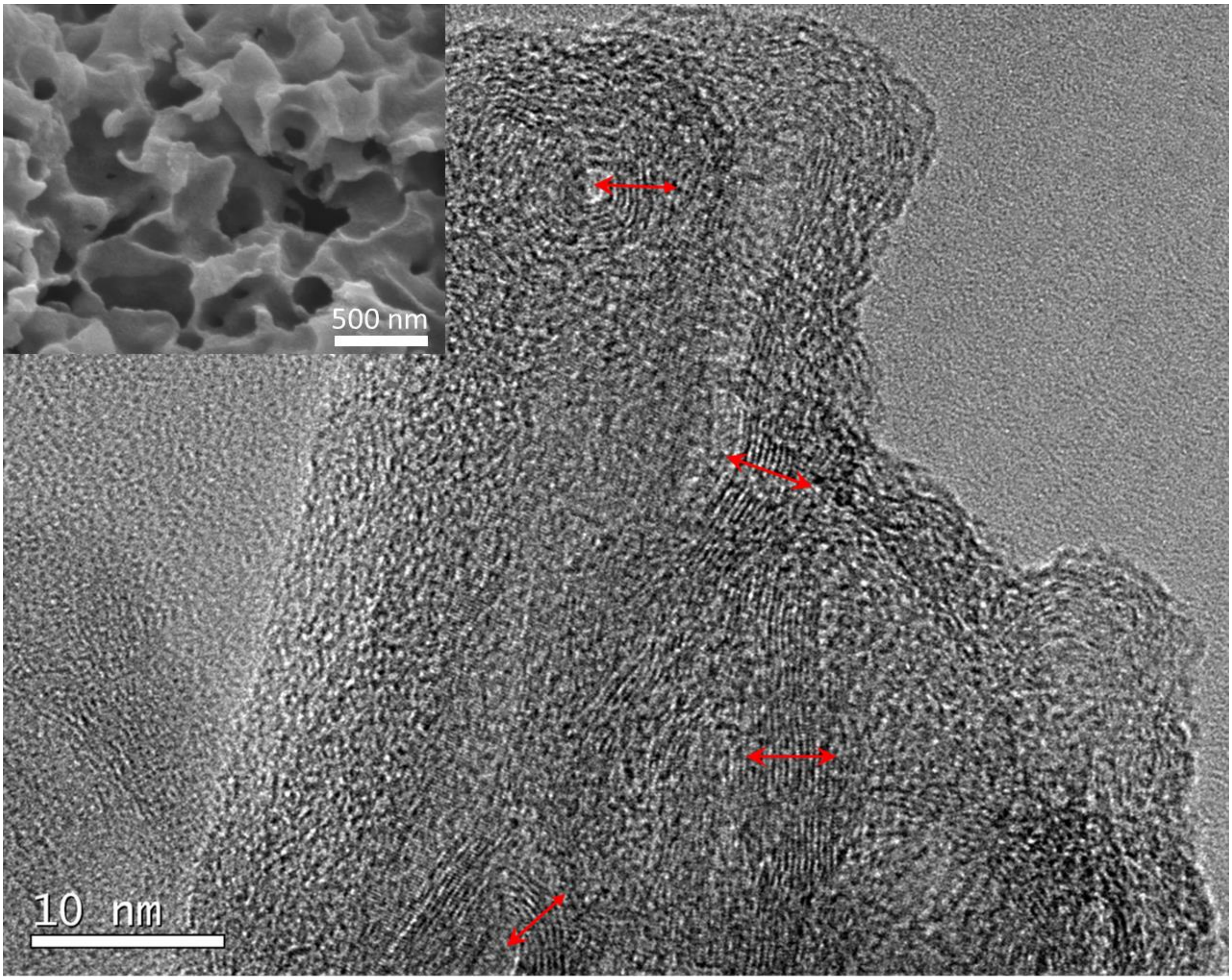

Figure S2. High resolution TEM (HRTEM) image of the PCN-NaCA-2.

Notes: the image clearly shows the corrugated carbon nitride thin layers with a thickness of around 5 nm. Inset, SEM image PCN-NaCA-2, showing sheet-like morphology.

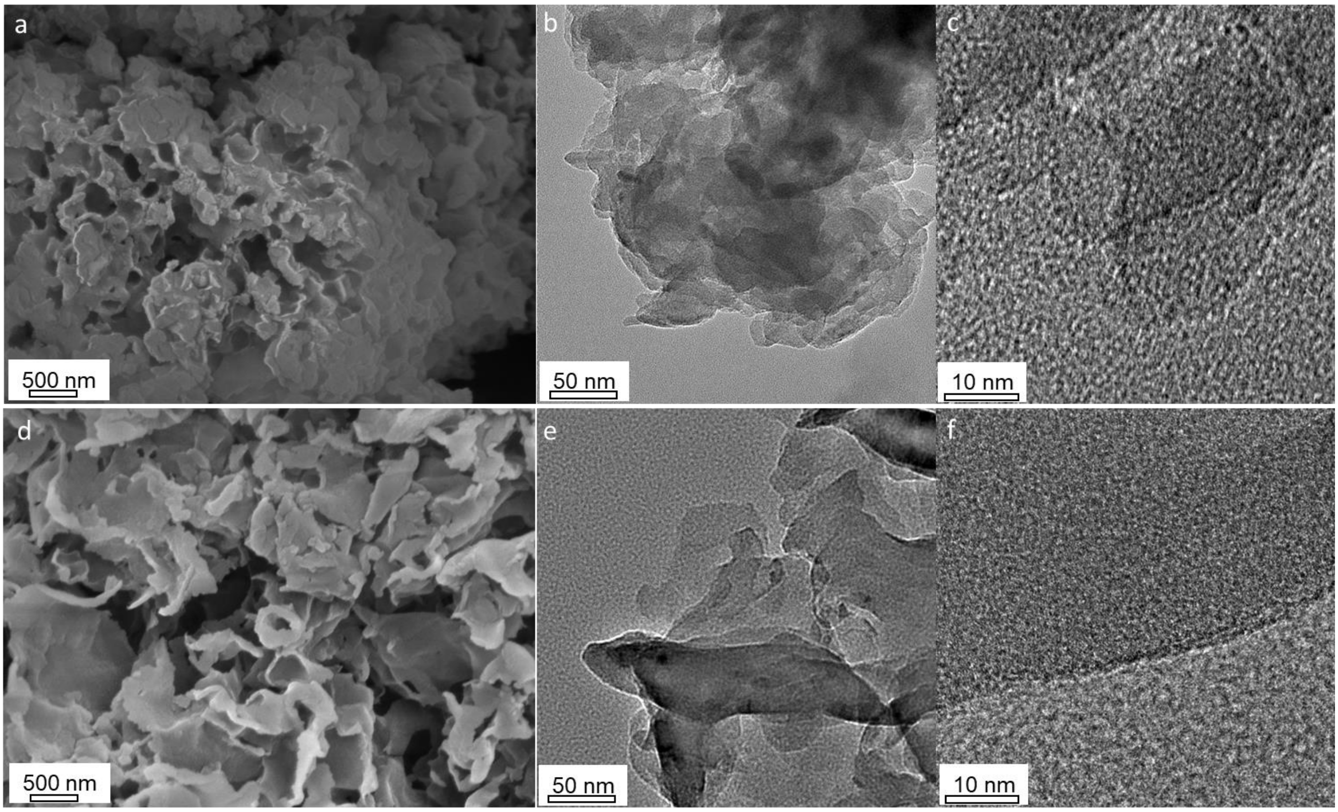

Figure S3. SEM (**a, d**) and HRTEM (**b**, **c**, **e**, and **f**) images of PCN-NaCA-1 (**a**, **b**, **c**) PCN-NaCA-3 (**d**, **e**, **f**).

Notes: SEM images shows that these samples have similar morphology; and due to the poor crystallinity of these samples, layer-stackings structures are not observed by HRTEM.

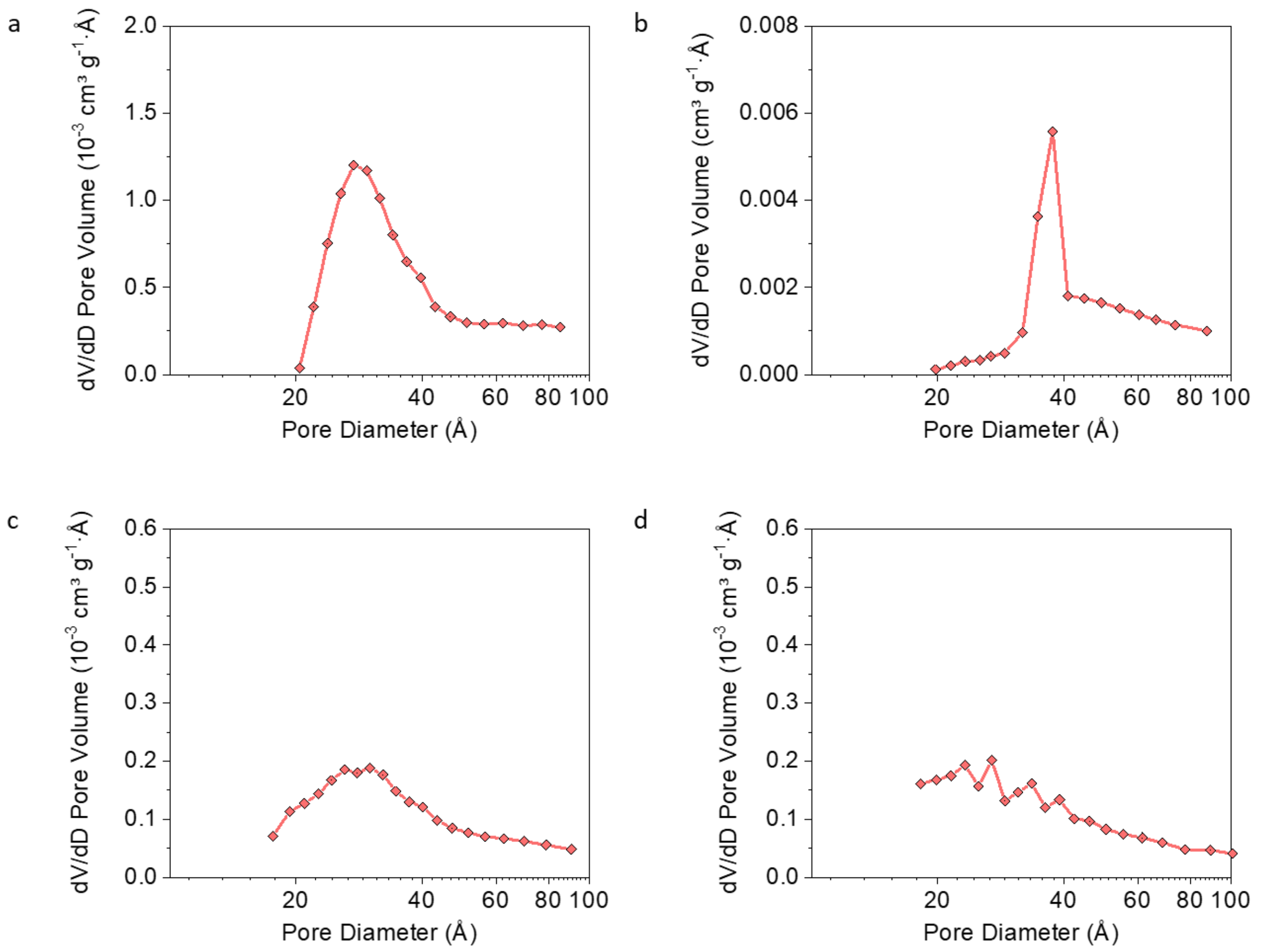

Figure S4. BJH Pore size distribution. **a** PCN; **b** PCN-NaCA-1; **c** PCN-NaCA-2; **d** PCN-NaCA-3.

Table S1. BET surface area of the samples.

Sample	BET surface area ($m^2\,g^{-1}$)
PCN	83.2
PCN-NaCA-1	95.6
PCN-NaCA-2	11.9
PCN-NaCA-3	18.1

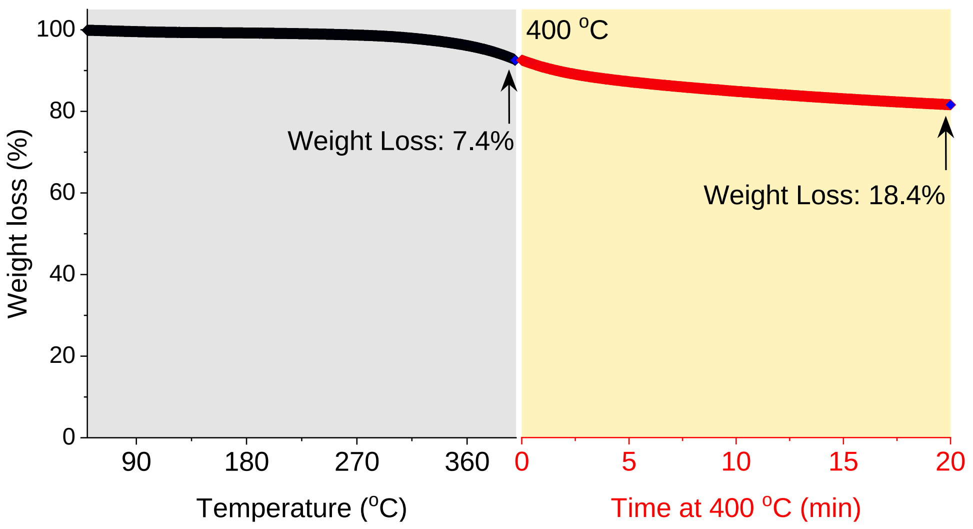

Figure S5. Thermal gravimetric analysis (TGA) curve from the PCN-NaCA-2 synthesis process monitored by thermal gravimetric analyzer-gas chromatograph-mass spectrometer (TGA-GC-MS). The precursor (PCN and NaSCN) for PCN-NaCA-2 was heated under nitrogen flow in an alumina crucible of the thermal gravimetric analyzer. Temperature program: initial temperature, 45 °C; ramp rate, 15 °C min^{-1} to 400 °C; final temperature, 400 °C hold for 20 min.

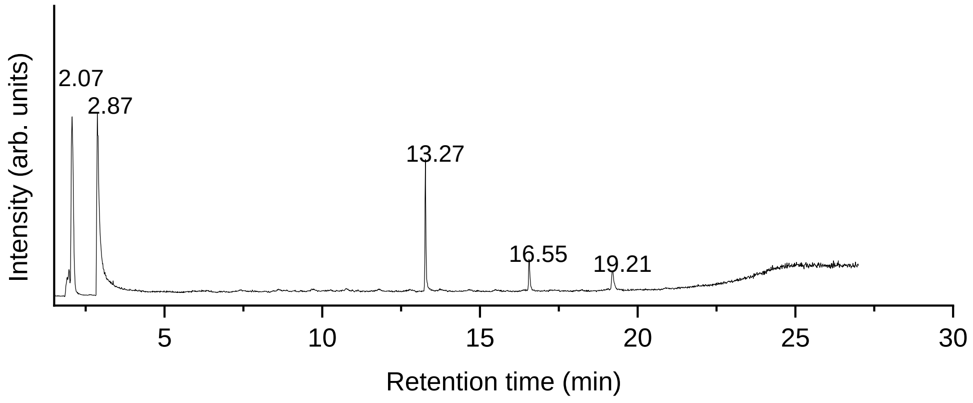

Figure S6. Chromatogram of the evolved gas at 400 °C during PCN-NaCA-2 synthesis process monitored by thermal gravimetric analyzer-gas chromatograph-mass spectrometer (TGA-GC-MS).

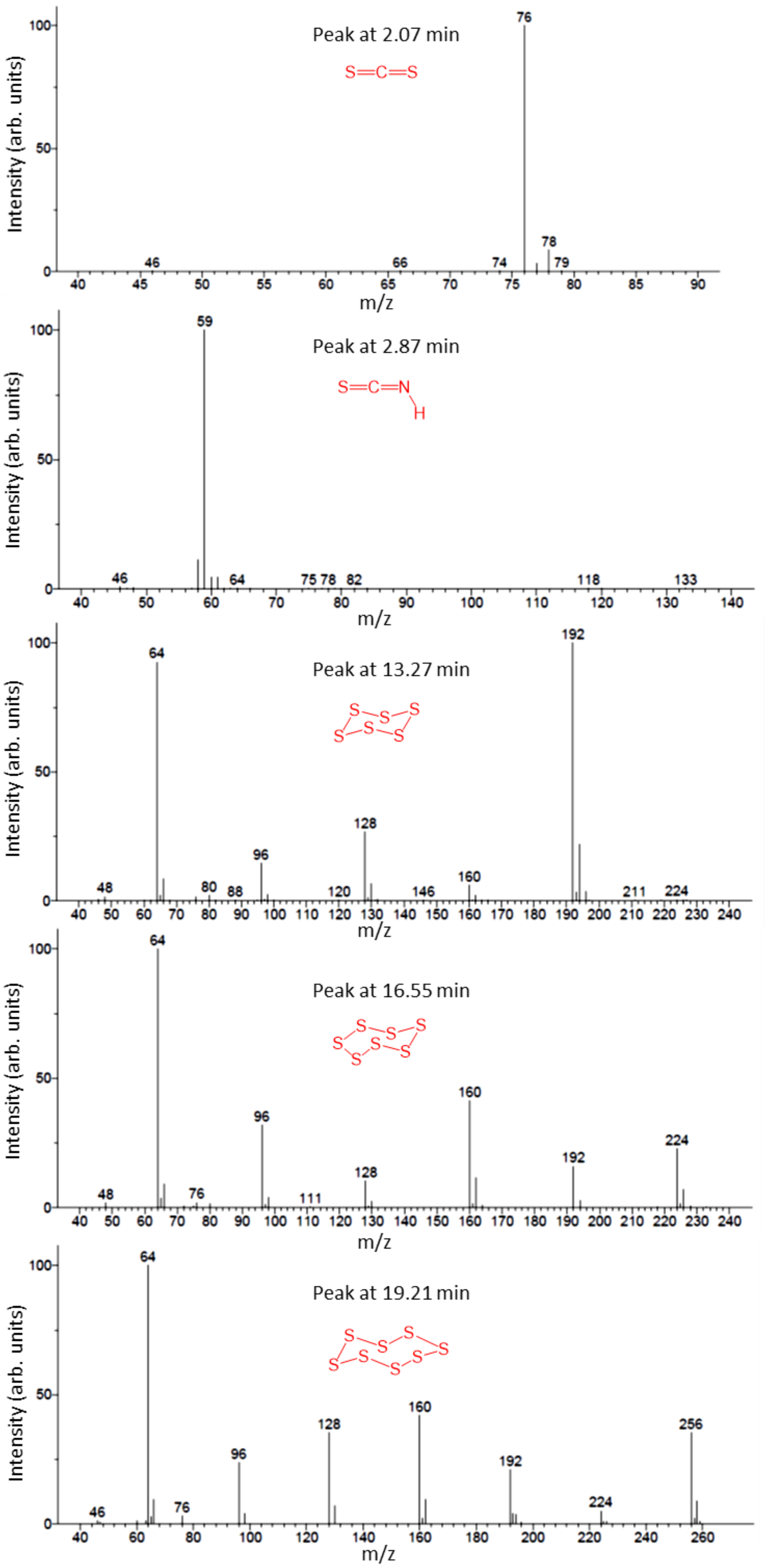

Figure S7. The mass spectra of every retention peaks in the chromatogram in Figure S6.

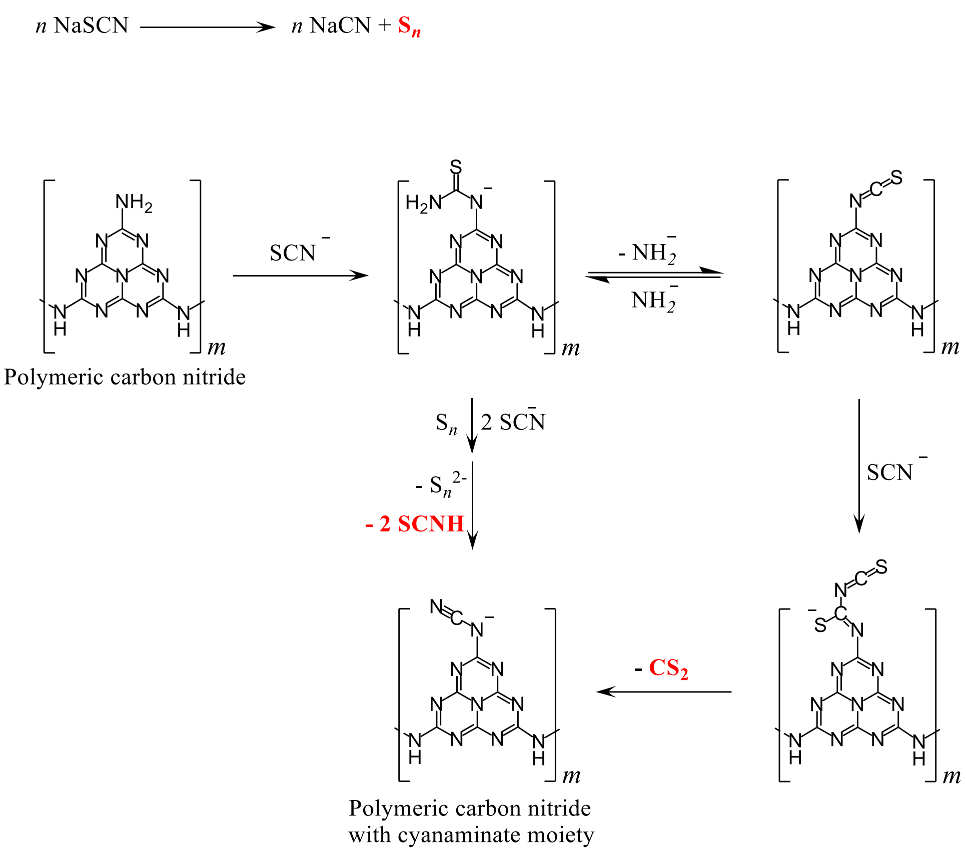

Figure S8. Proposed reaction mechanism of the cyanaminate moiety formation based on the experimentally confirmed species (S_n, CS_2, and SCNH) in TG-GCMS characterization as well as the discussions from Sattler and Schnick.[23]

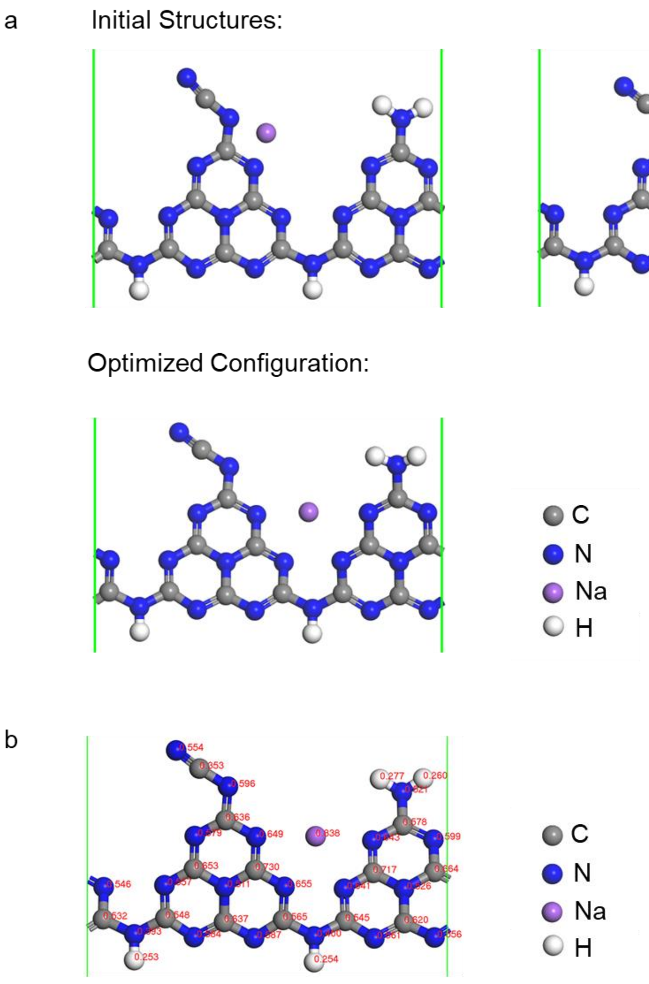

Figure S9. **a** Initial structures and the optimized configuration PCN-NaCA; **b** the Mulliken charge population of PCN-NaCA with one extra electron.

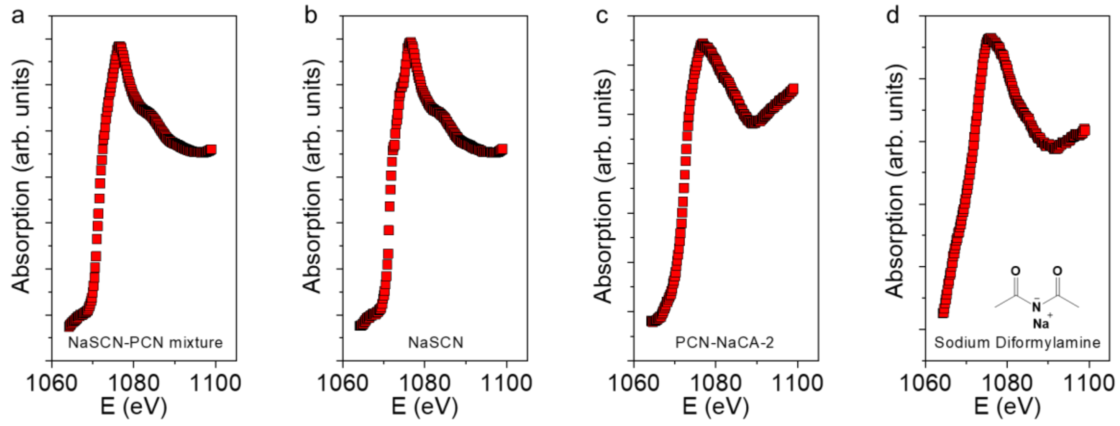

Figure S10 Na K-edge x-ray absorption spectra. **a** NaSCN (20 wt.%) / PCN prepared via Incipient wetness impregnation; **b** Sodium thiocyanate; **c** PCN-NaCA-2; **d** Sodium diformylamine.

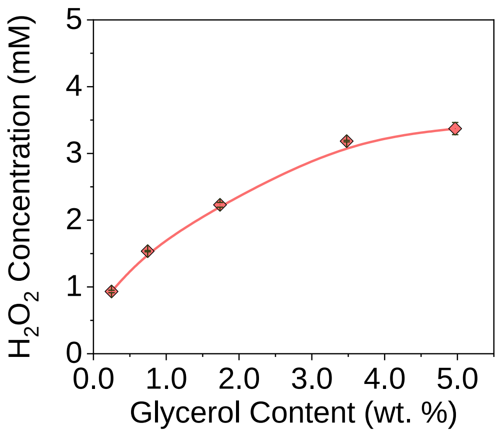

Figure S11. Photocatalytic H_2O_2 production performance of PCN-NaCA-2 in the aqueous solution with various glycerol contents. Reaction conditions: 10 mg photocatalyst and 50 mL aqueous solution with various glycerol contents was charged in the photoreactor and irradiated with solar simulator for 1 hour. The error bars are the standard deviations from the mean value of H_2O_2 concentrations in triplicate reactions.

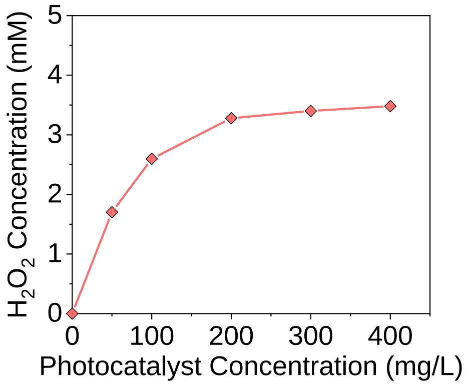

Figure S12. Photocatalytic H_2O_2 production performance of PCN-NaCA-2 in 50 mL batch photo-reactor with various photocatalyst concentrations. Reaction conditions, 50 mL aqueous suspension with PCN-NaCA-2 and 3.5 wt.% glycerol, 1 atm. O_2, irradiation with a solar simulator (100 mW cm^{-2} on the outer-surface of the jacketed photo-reactor) for 1 hour.

Table S2. Selected recently reported photocatalytic systems with efficient solar hydrogen peroxide production performance.

Photocatalysts	Protons/Electrons Donor	Irradiation Conditions	H_2O_2 Production Performance	Reference
PCN-NaCA-2	Glycerol (3.5 wt.%)	Solar simulator	18.7 μmol h^{-1} mg^{-1}_{Catal}<br>AQY: 380 nm, 27.6 %; 420 nm, 11.8%.	This work
Na-K-S-C_3N_4	Ethanol (10 wt.%)	λ ≥ 420 nm, 100 mW cm^{-2}	2.7 μmol h^{-1} mg^{-1}_{Catal}	*24*
K,Na-PHI	Glycerol (1 wt.%)	λ = 365nm, 40 mW cm^{-2}	0.35 μmol h^{-1} mg^{-1}_{Catal}	*25*
	Ethanol (1 wt.%)		0.87 μmol h^{-1} mg^{-1}_{Catal}	
TAPD-$(Me)_2$ COF	Ethanol (10 wt.%)	λ: 420 - 700 nm	0.23 μmol h^{-1} mg^{-1}_{Catal}	*26*
PM-CDs	H_2O	λ≥ 420 nm, 34.8 mW cm^{-2}	1.8 μmol h^{-1} mg^{-1}_{Catal}<br>AQY: 630 nm, 0.54 %	*27*
Resin	H_2O	λ ≥ 420 nm, 14.0 mW cm^{-2}	0.08 μmol h^{-1} mg^{-1}_{Catal}<br>(~ 99 μmol H_2O_2 produced from 24h reaction)<br>Solar to chemical conversion: > 0.5%	*28*
MIL-125-R7	Benzyl alcohol (Benzyl Alcohol/Water two phase)	λ ≥ 420 nm	0.32 μmol h^{-1} mg^{-1}_{Catal}	*29*
OCN-500	Isopropanol (10 wt.%)	λ ≥ 420 nm, 35.2 mW cm^{-2}	2.92 μmol h^{-1} mg^{-1}_{Catal}<br>AQY: 365 nm, 28.5 %; 420 nm,10.2 %.	*30*
	H_2O		0.11 μmol h^{-1} mg^{-1}_{Catal}	
g-C_3N_4-PDI-$rGO_{0.05}$	H_2O	λ ≥ 420 nm, 13.1 mW cm^{-2}	0.08 μmol h^{-1} mg^{-1}_{Catal}<br>(~ 40 μmol H_2O_2 produced from 2 h reaction)<br>Solar to chemical conversion: 0.2%	*31*
g-C_3N_4-PDI	Isopropanol (Pure)	λ: 420 - 500 nm, 2.69 mW cm^{-2}	0.70 μmol h^{-1} mg^{-1}_{Catal}	*32*
	H_2O		0.02 μmol h^{-1} mg^{-1}_{Catal}	

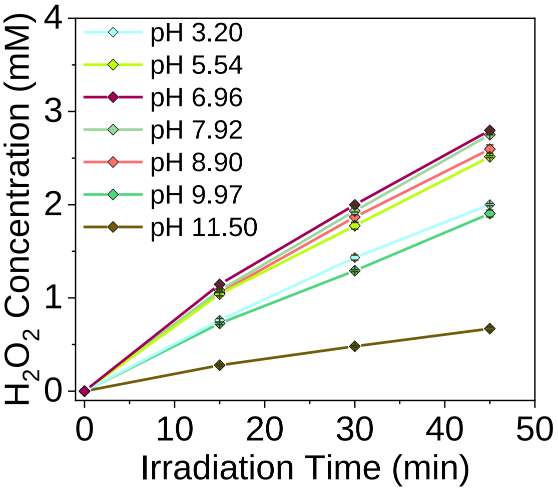

Figure S13. The impact of the initial pH value of the suspension on the performance of PCN-NaCA-2 in the photocatalytic H_2O_2 production. Reaction conditions, 50 mL aqueous suspension containing 10 mg PCN-NaCA-2 and 3.5 wt.% glycerol, 1 atm. O_2, irradiation with a solar simulator (100 mW cm^{-2} on the outer-surface of the jacketed photo-reactor). The error bars are the standard deviation from the mean value of H_2O_2 production in triplicate experiments.

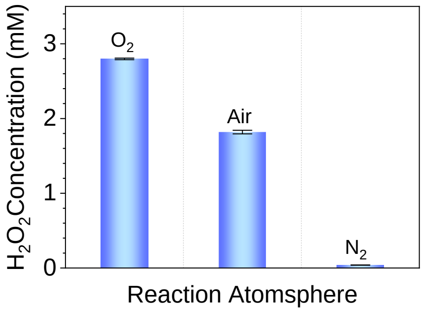

Figure S14. Comparison of the photocatalytic H_2O_2 production performance in O_2, air, and N_2 atmosphere. Reaction conditions, the same to that in figure S13, except that the atmosphere is controlled. Reaction conditions: 10 mg photocatalyst, 50 mL aqueous solution with glycerol concentration of 3.5 wt.% was mixed by ultrasonication and irradiated by solar simulator for 45 min. The error bars are the standard deviations from the mean value of H_2O_2 production in triplicate experiments.

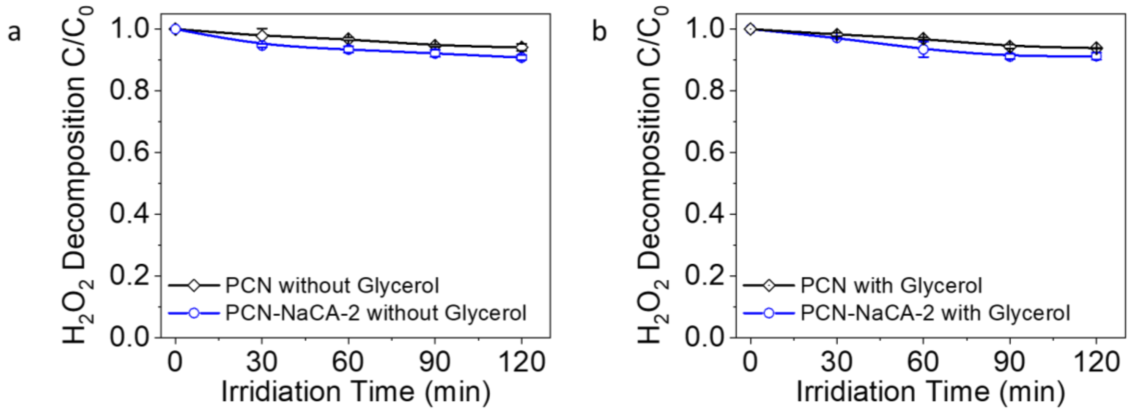

Figure S15. Photocatalytic decomposition of H_2O_2 on PCN and PCN-NaCA-2 in the absence (**a**) and in the presence of glycerol (**b**). Reaction conditions: 5 mg photocatalyst and 35 mL H_2O_2 solution with concentration of 6.5 mM was charged in a 50 mL photoreactor. The photoreactor was sealed and the oxygen in the reactor was removed by repeated vacuum and nitrogen refilling. The oxygen free reaction mixture was then irradiated with solar simulator. The error bars are the standard deviations from the mean values from triplicate experiments.

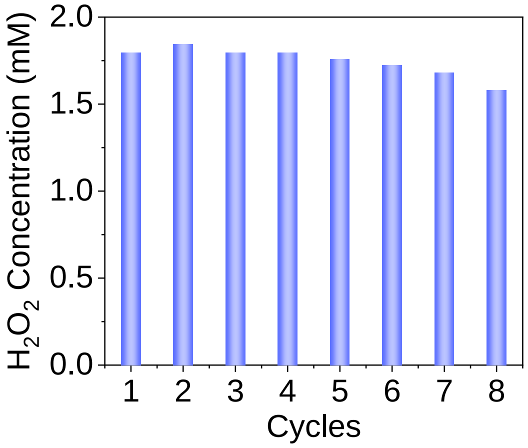

Figure S16. Cycle test of PCN-NaCA-2 in the photocatalytic H_2O_2 production. Reaction conditions: 20 mg PCN-NaCA-2 was added in the 50 mL aqueous solution with 3.5 wt.% glycerol in a 50 mL jacketed photo-reactor at room temperature of 25 °C. The reaction mixture was irradiated by solar simulator for 30 min for each cycle. After the reaction, the photocatalyst was separated by centrifugation, and 50 mL fresh aqueous glycerol solution (3.5 wt.%) was charged into the photoreactor with the recycled photocatalyst for the next run.

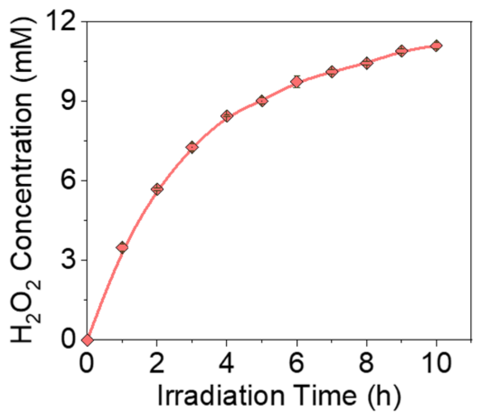

Figure S17. Long term running performance of PCN-NaCA-2 in photocatalytic H_2O_2 production. Reaction conditions: 20 mg photocatalyst was dispersed in 50 mL aqueous solution with glycerol contents of 3.5 wt.% was charged in the photoreactor and irradiated with solar simulator. The error bars are the standard deviations from the mean values from triplicate experiments.

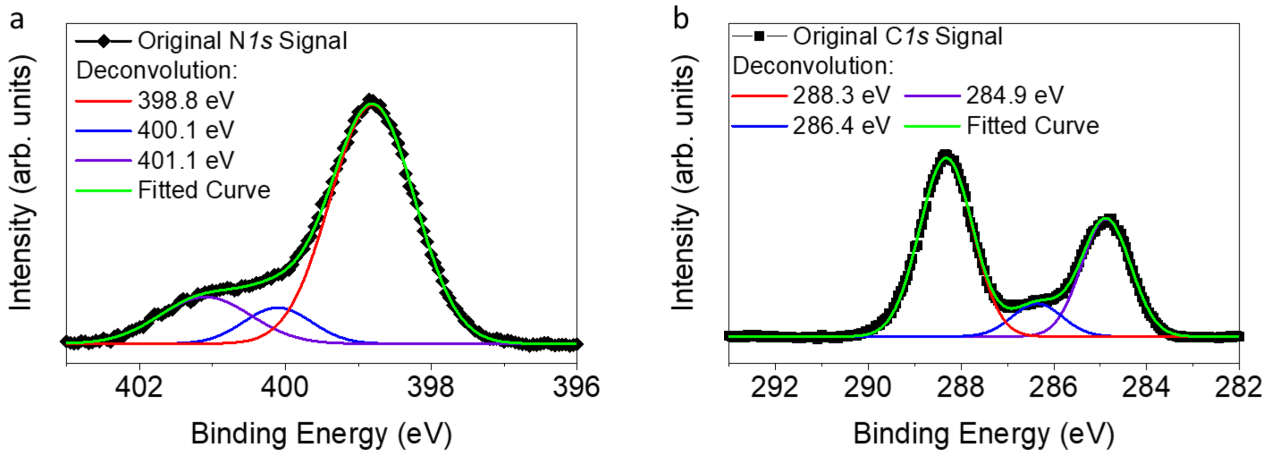

Figure S18. XPS characterization of PCN-NaCA-2 recycled after 10 hours photocatalytic H_2O_2 production.

Notes: for N*1s* signal, the deconvoluted peaks at 400.1 eV decreases as compared to that of as-prepared PCN-NaCA-2, which might result from the changes in the amine moiety of PCN-NaCA-2 after long-term running under irradiation; for C*1s* signal, there is no obvious changes except that the increased peak intensity at 284.9 eV, which could be attributed to the surface deposited adventitious carbon after the reactions involving organics. The sodium content after long-term irradiation was determined to be 1.8 wt.% by ICP-OES.

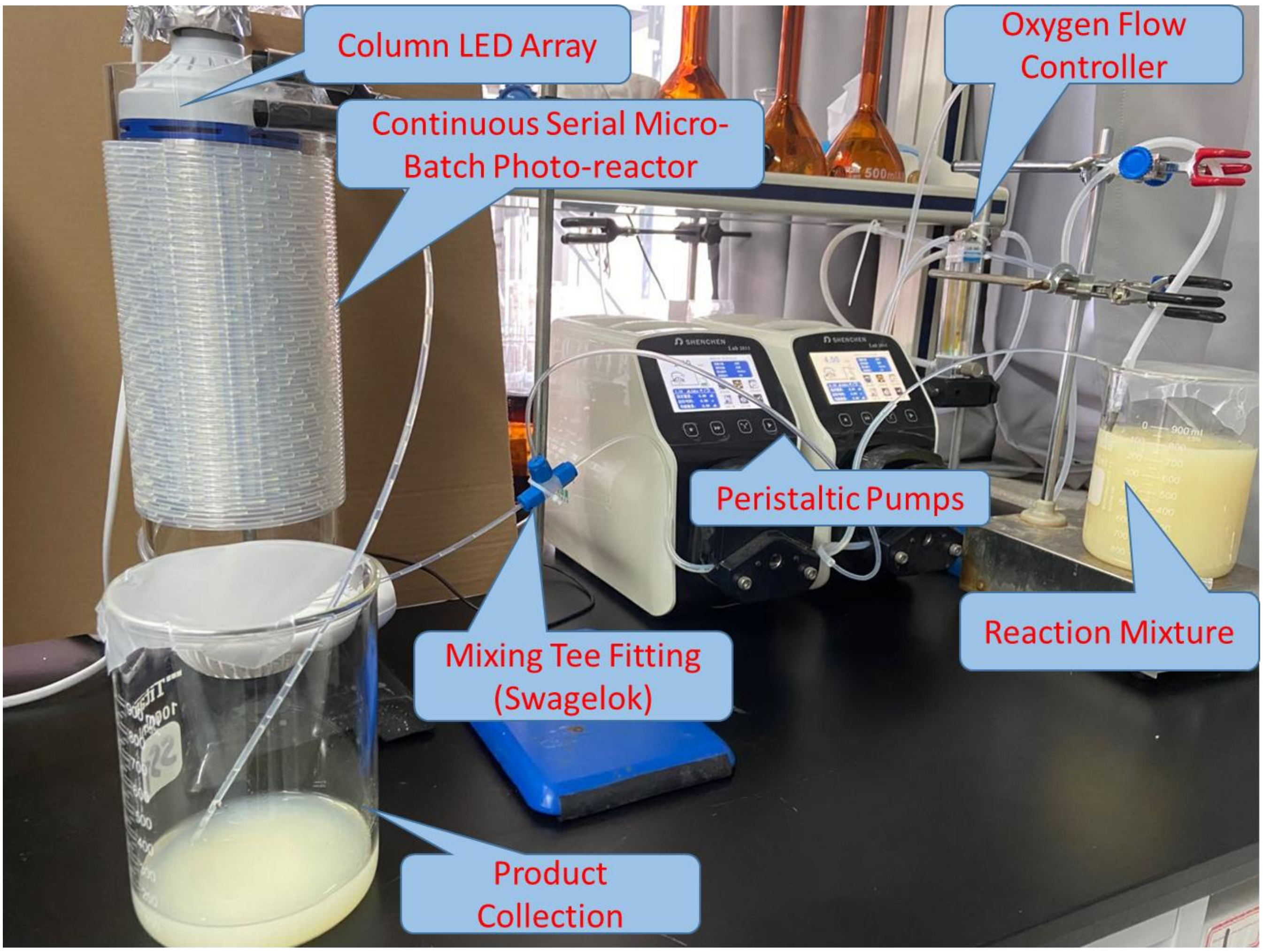

Figure S19. Photograph of the continuous serial micro-batch photo-reactor.

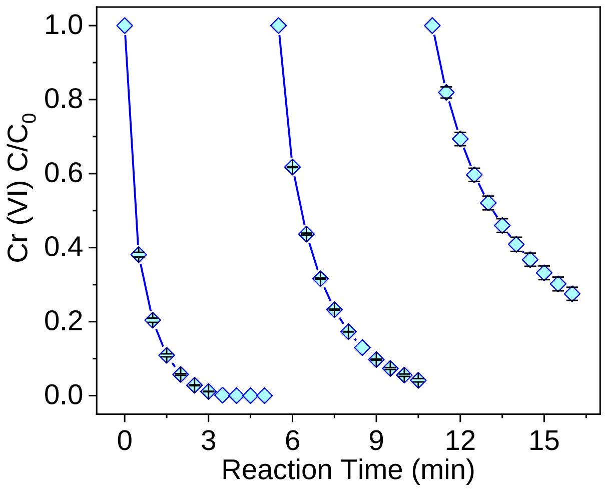

Figure S20. Reductive elimination of Cr(VI) by photocatalytically generated hydrogen peroxide. Reaction conditions: 1.8 mL H_2O_2 solution with concentration of 10 mmol produced by PCN-NaCA-2 photocatalysis was charged into a silica cuvette. 200 uL of Cr(VI) solution with concentration of 400 ppm was added in the cuvette and mixed by magnetic stirrer. The concentration of Cr(VI) was determined by the absorbance of the solution at 340 nm with a calibration curve. The error bars are the standard deviation from the mean values of Cr(VI) concentrations from triplicate experiments.

Notes:

Reductive conversion of the highly toxic hexavalent Cr to less toxic trivalent Cr is one of the most important approaches for treating hexavalent Cr contamination. [33,34] Hydrogen peroxide is an efficient and environmental-benign reductant for hexavalent Cr conversion:

$$2\ HCrO_4^- + 3\ H_2O_2 + 8\ H^+ = 2\ Cr(III) + 3\ H_2O + 8\ O_2 \qquad (7)$$

The photocatalytically generated H_2O_2 solution was applied in the Cr(VI) reduction reaction. Cr(VI) with a concentration of 40 ppm was eliminated within 3 min. Addition of Cr(VI) stock solution in to the reaction system led to a spike in Cr(IV) concentration to 40 ppm, and 5 min reaction reached 95% Cr(VI) removal. After the third spike in Cr(VI) concentration to 40 ppm, 5 min reaction reached 72% Cr(VI) removal. These tests implicate the potential application of the photocatalytically generated H_2O_2 solution for the environmental Cr(VI) pollution elimination.

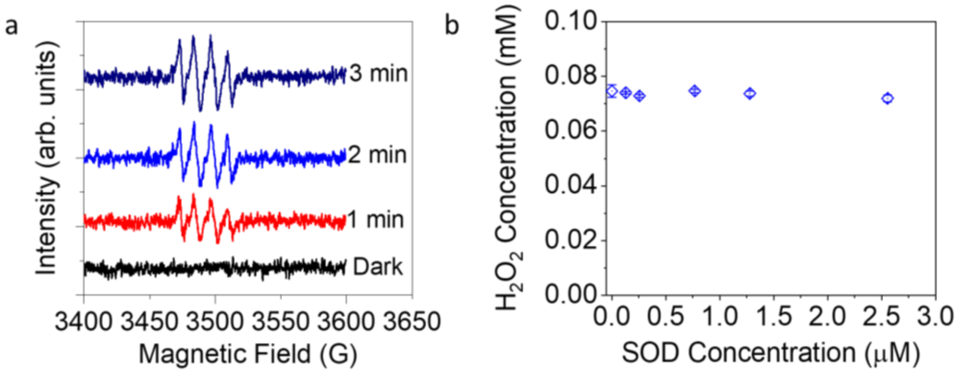

Figure S21. a DMPO (5,5-Dimethyl-1-pyrroline N-oxide) – superoxide radical adduct signal monitored by ESR (Electron Spin Resonance) at various reaction times on PCN-NaCA-2 in oxygen saturated CH_3CN/CH_3OH (v/v, 10/1) solvent; **b** Examination of the contribution of superoxide radical disproportionation on the H_2O_2 production by SOD (superoxide dismutase) probe reaction. Reaction conditions: PCN-NaCA-2 (0.2 mg mL^{-1}), 2 mL phosphate buffer solution (1 mM, pH 7.4) with 3.5 wt.% glycerol and various SOD concentration was charged in a photoreactor; the oxygen-saturated reaction mixture was irradiated with 420 nm LED for 1 min and then sampled for H_2O_2 concentration analysis. The error bars in (**b**) are the standard deviations from the mean values of H_2O_2 production in triplicate experiments.

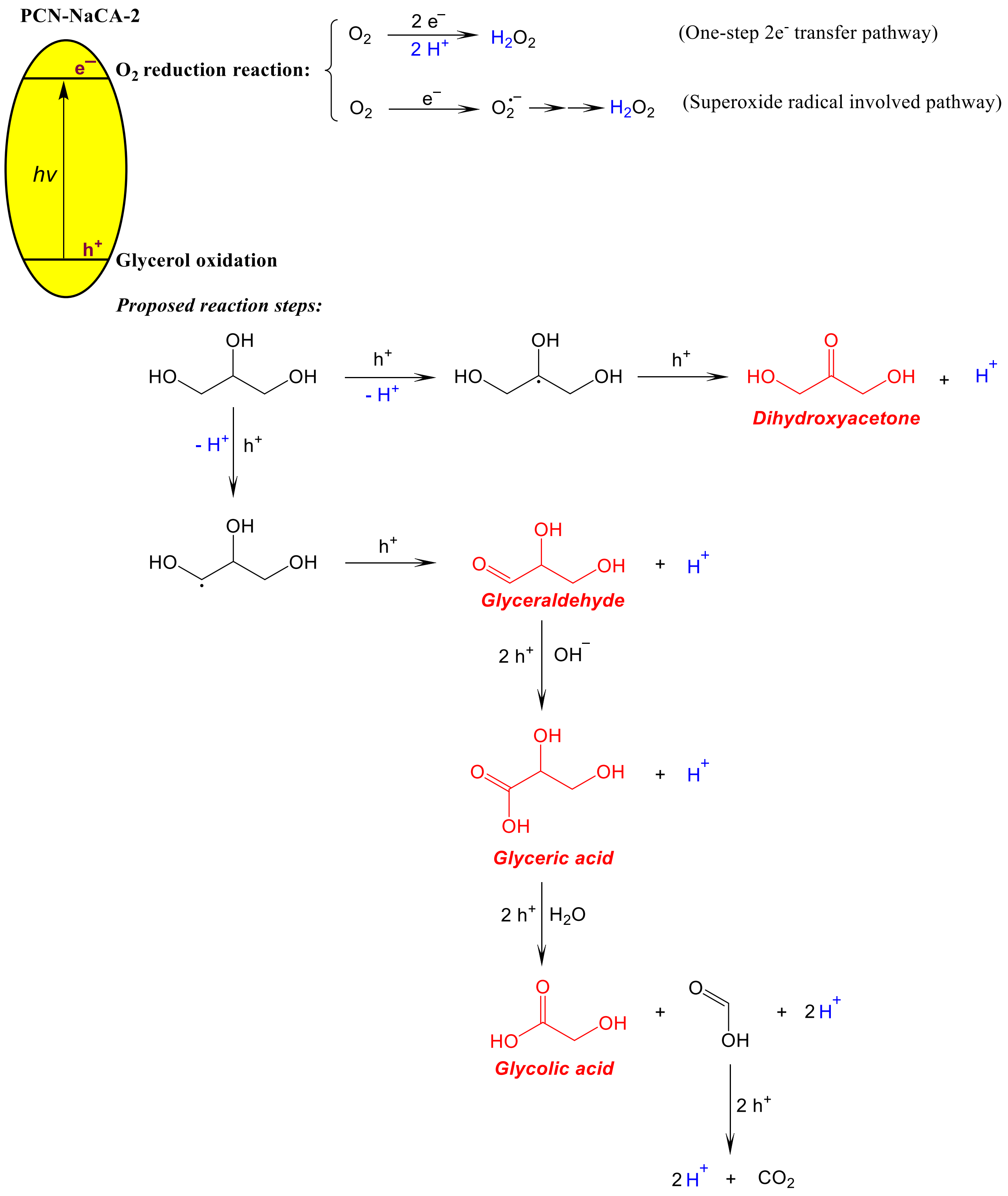

Figure S22. Scheme of the proposed reaction mechanism of dioxygen reduction and glycerol degradation. The glycerol oxidation reaction mechanism was proposed based on the GC-MS identified intermediates, e.g., dihydroxylacetone, glyceraldyhyde, glyceric acid, and glycolic acid.

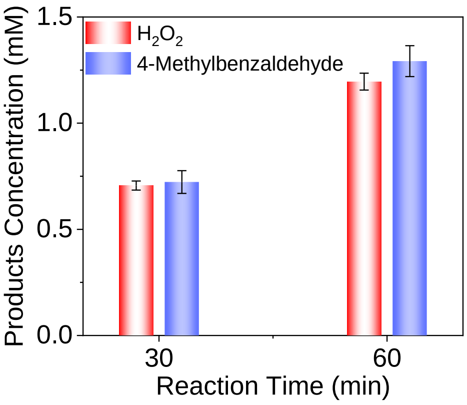

Figure S23. Photocatalytic H_2O_2 production with 4-methylbenzyl alcohol as the electron/proton donor on PCN-NaCA-2 under 420 nm irradiation. Reaction conditions: 20 mg PCN-NaCA-2 was dispersed in 10 mL acetonitrile with 1.5 g 4-methylbenzyl alcohol and 0.15 g H_2O. The photoreactor was filled with 1 atm. O_2, capped, and irradiated by a LED lamp (λ = 420 nm, 25.0 mW cm^{-2}). 4-Methylbenzaldehyde was quantified by GCMS with calibration curve and 1,4-dicyanobenzene as the internal standards. The error bars are the standard deviations from the mean values of product concentrations from triplicate experiments.

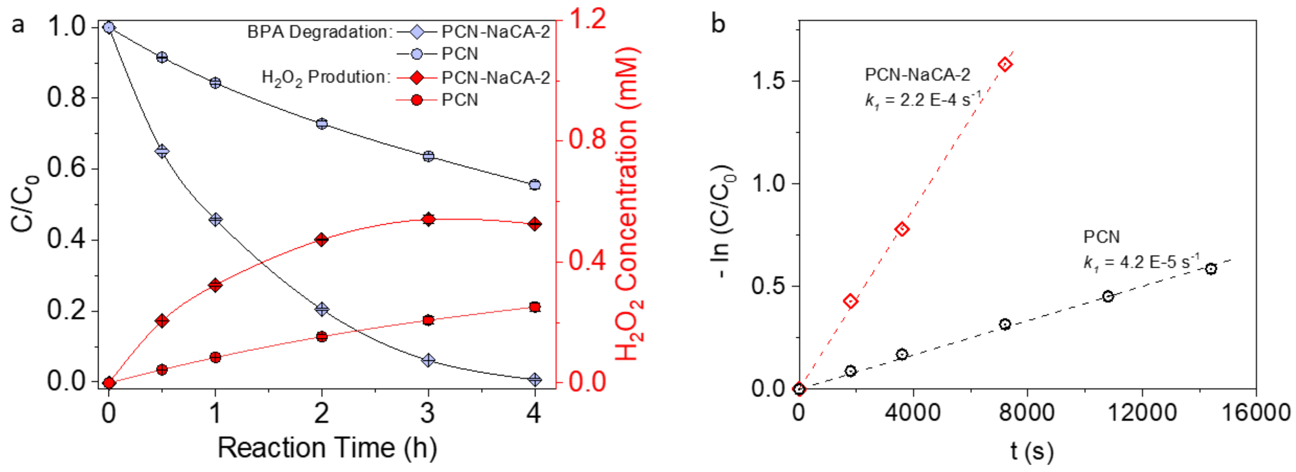

Figure S24. a Photocatalytic H_2O_2 production coupled with bisphenol A (BPA) degradation; **b** pseudo first-order kinetic plots of the photocatalytic BPA degradation. Reaction conditions: 10 mg photocatalyst was dispersed in 50 mL BPA aqueous solution with concentration of 100 ppm by ultrasonication. The photocatalytic reaction was conducted in a jacketed-photoreactor. The light source was a solar simulator with intensity of 100 mW cm^{-2} on the surface of the jacketed reactor. The error bars (**a**) are the standard deviation from the mean values from triplicate experiments.

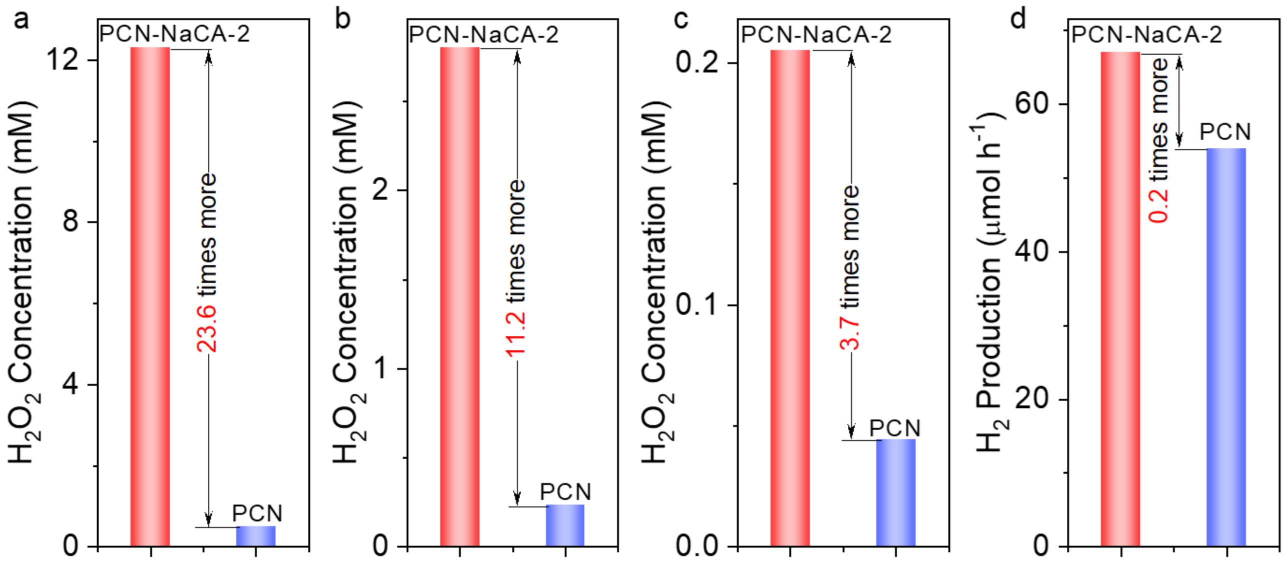

Figure S25. Comparison of PCN and PCN-NaCA-2 in different reactions. **a** Photocatalytic H_2O_2 production in the flow reactor under sufficient photons irradiation in the presence of glycerol as the proton/electron donor; **b** photocatalytic H_2O_2 production in the batch reactor in the presence of glycerol as the proton/electron donor; **c** photocatalytic H_2O_2 production in the batch reactor in the presence of bisphenol A (BPA) as the proton/electron donor; **d** photocatalytic H_2 evolution in anaerobic condition.

Table S3. PL Lifetime of photo-induced charge carriers and their relative contribution in PCN and PCN-NaCA-2.

Sample	τ_1	A_1	τ_2	A_2	τ_{avg} (ns)
PCN	1.35	0.447	9.10	0.553	8.27
PCN-NaCA-2	0.88	0.600	4.26	0.400	3.46

Notes: the samples were characterized by the time resolved photoluminescence spectroscope with a single photon picosecond laser at a wavelength of 355 nm. The PL decay curves was fitted exponentially using the following equation:

$$I(t) = A_1 e^{-t/\tau_1} + A_2 e^{-t/\tau_2} \quad (8)$$

where, A_1 and A_2 represent the normalized amplitudes of each decay component, and τ_1 and τ_2 are values of the lifetime components, respectively. The average life time is calculated based on the following equation:

$$\tau_{avg} = (A_1\tau_1^2 + A_2\tau_2^2)/(A_1\tau_1 + A_2\tau_2) \quad (9)$$

For carbon nitride, polymerization reaction continues in the molten salt, and the polymerization degree can be improved, as shown in XRD/HR-TEM data in Figures S1 and S2, and the reference.[35] Increased polymerization degree leads to extended π-conjugated systems and delocalized π-electrons, and thus enhances the charge migration rate.[36] Therefore, on PCN-NaCA-2, the photo-induced charges can move to the trapping sites more efficiently, which results in the short PL decay lifetime.[37]

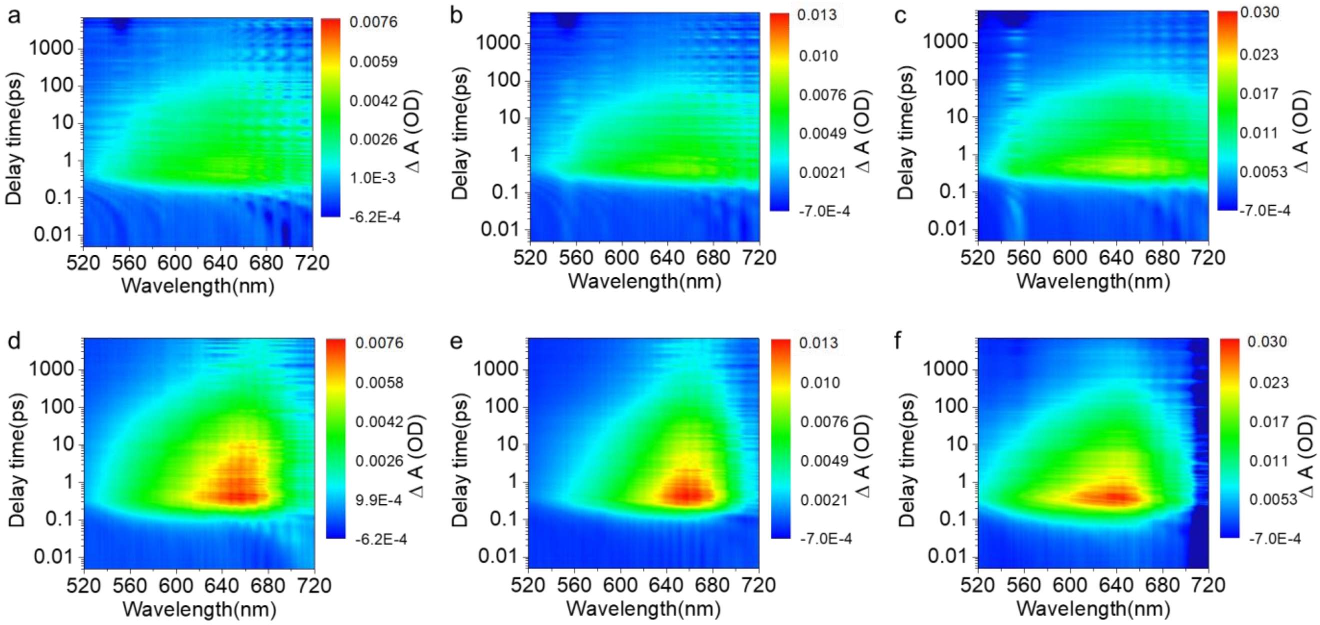

Figure S26. Femtosecond transient absorption spectra (fs-TAS) of PCN-NaCA-2 after excitation by 365 nm laser pulse with energy density of 35.8 (**a**, **d**), 79.6 (**b**, **c**), 278.7 (**c**, **f**) μJ cm^{-2} in glycerol aqueous solution (3.5 wt.%) under vacuum (**a**, **b**, and **c**) and in 1 atm. oxygen atmosphere (**d**, **e**, and **f**).

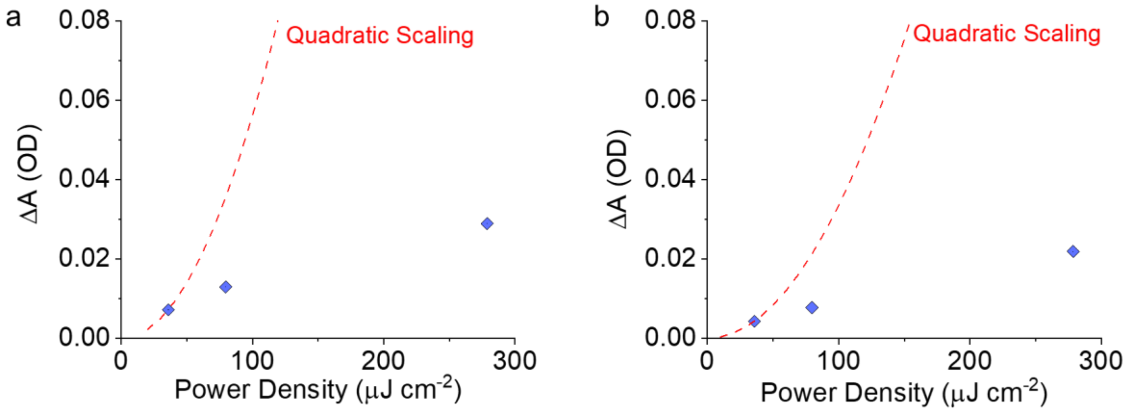

Figure S27. The relevance of the power densities to the theoretical quadratic scaling. **a** with dioxygen; **b** under vacuum.

Notes: the data plots significantly deviate from a quadratic scaling as would be expected for two-photon excitation, indicating that two-photon excitation is not relevant to the power densities used in our experiment.

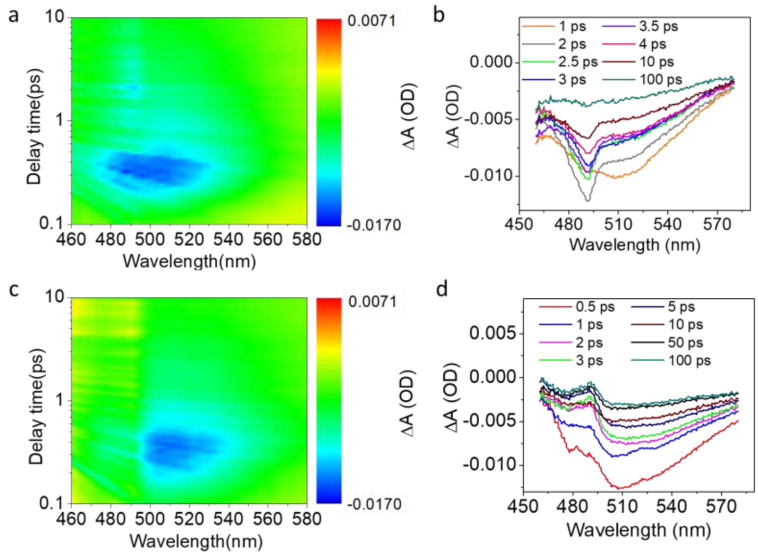

Figure S28. Femtosecond transient absorption spectra (fs-TAS) of PCN after excitation by 365 nm laser pulse with energy density of 278.7 μJ cm^{-2} in glycerol aqueous solution (3.5 wt.%) in oxygen (a, b) and under vacuum (c, d) conditions.

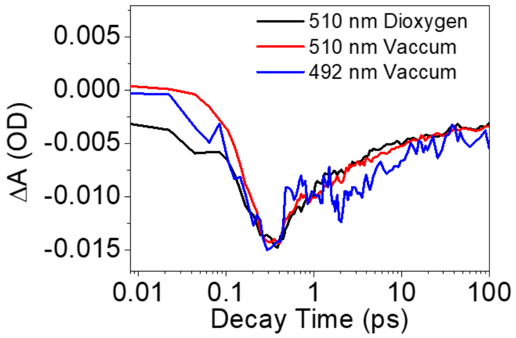

Figure S29. The decay kinetics of femtosecond transient absorption spectra (fs-TAS) bleach signal of the PCN after excitation by 365 nm laser pulse with energy density of 278.7 μJ cm^{-2} in glycerol aqueous solution (3.5 wt.%) in oxygen and under vacuum conditions.

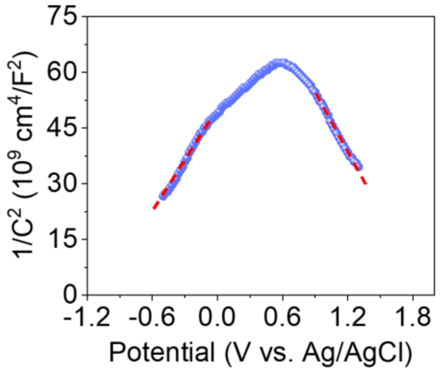

Figure S30. Mott-Schottky plots of PCN-NaCA-2 in dark at the frequency of 1000 Hz with a three-electrode system.

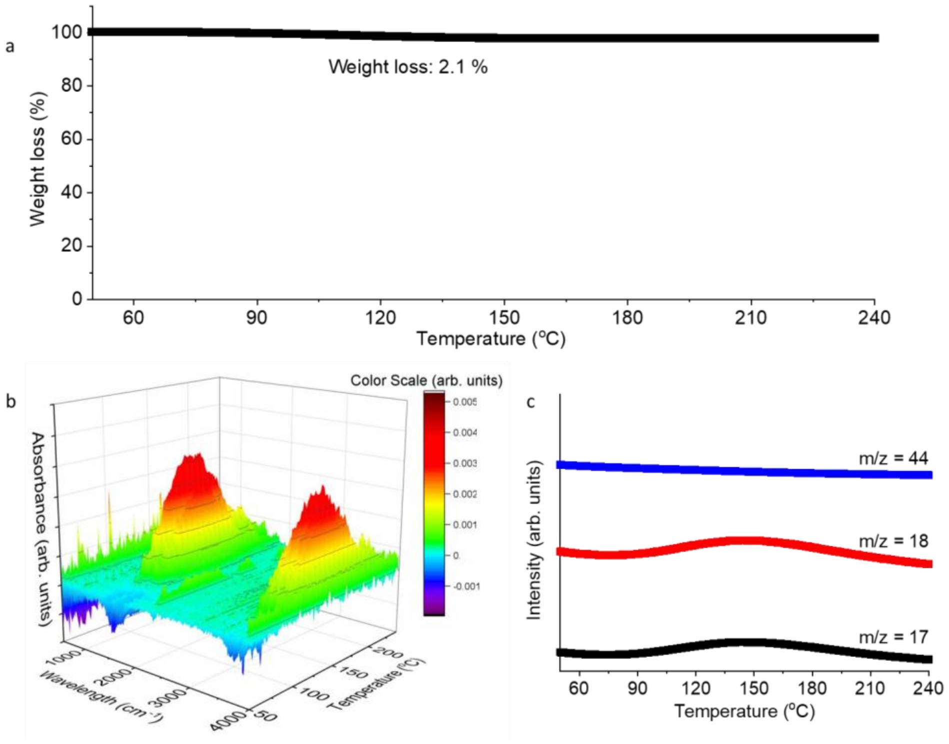

Figure S31. TG-IR-MS characterization of PCN-NaCA-2 in aerobic condition. **a** TG curve; **b** IR spectra of the evolved gases with temperature ramping; **c** MS signals of selected ions with rising temperature, CO_2 (m/z = 44) and H_2O (m/z = 17 and 18).

Notes: There is a small weight loss of 2.1 % in the TG curve. The infrared spectra observe the release of H_2O during heating, and the mass spectra also confirm that water is the only species evolved during heating. The evolved H_2O comes from the surface adsorption, since the sample was stored in air and did not pre-treated before measurement. These results indicate that pretreatment in helium flow at 300 ^oC is enough for removing the surface adsorbed molecules in O_2-TPD.

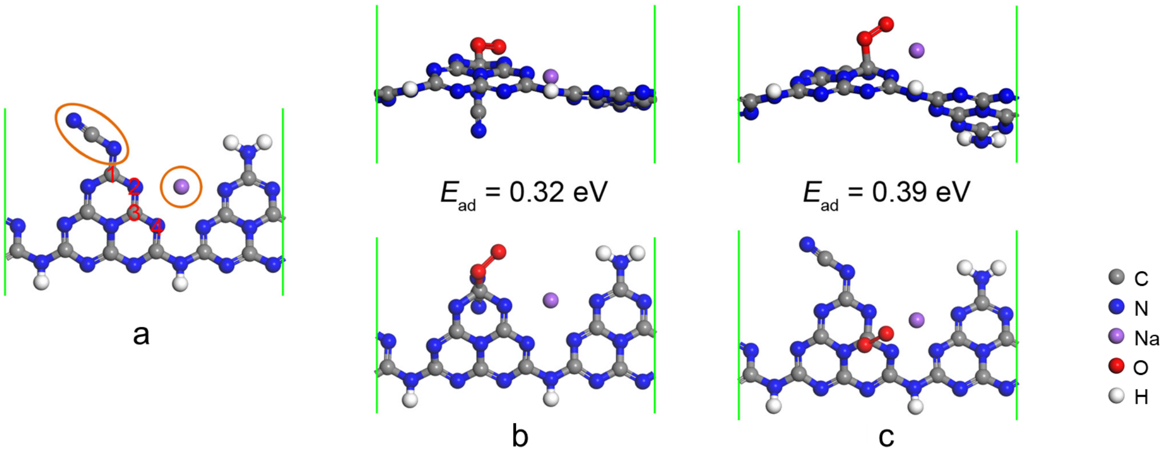

Figure S32. **a** Screening of O_2 adsorption sites closing cyanamino-group and sodium on PCN-NaCA. Optimized configurations of O_2 adsorption on sites 1 (**b**) and 3 (**c**).

Notes：Sodium cyanaminate moiety plays an essential role for the superior performance in the photocatalytic H_2O_2 production in the experimental investigations; and this work mainly focus on the mechanism behind the superior performance initiated by sodium cyanaminate moiety. The theoretical simulation is employed for further understanding the role of cyanaminate moiety in ORR. The sites closing to sodium and cyanamino-group (Sites 1 – 4) was thus screened for O_2 adsorption. It was found that O_2 is unable to adsorb on sites 2 and 4, but the adsorption is feasible on sites 1 and 3 with E_{ad} (adsorption energy) of 0.32 eV and 0.39 eV, respectively. O_2 adsorption on site 3 was selected as the optimum initial structure for the following calculation.

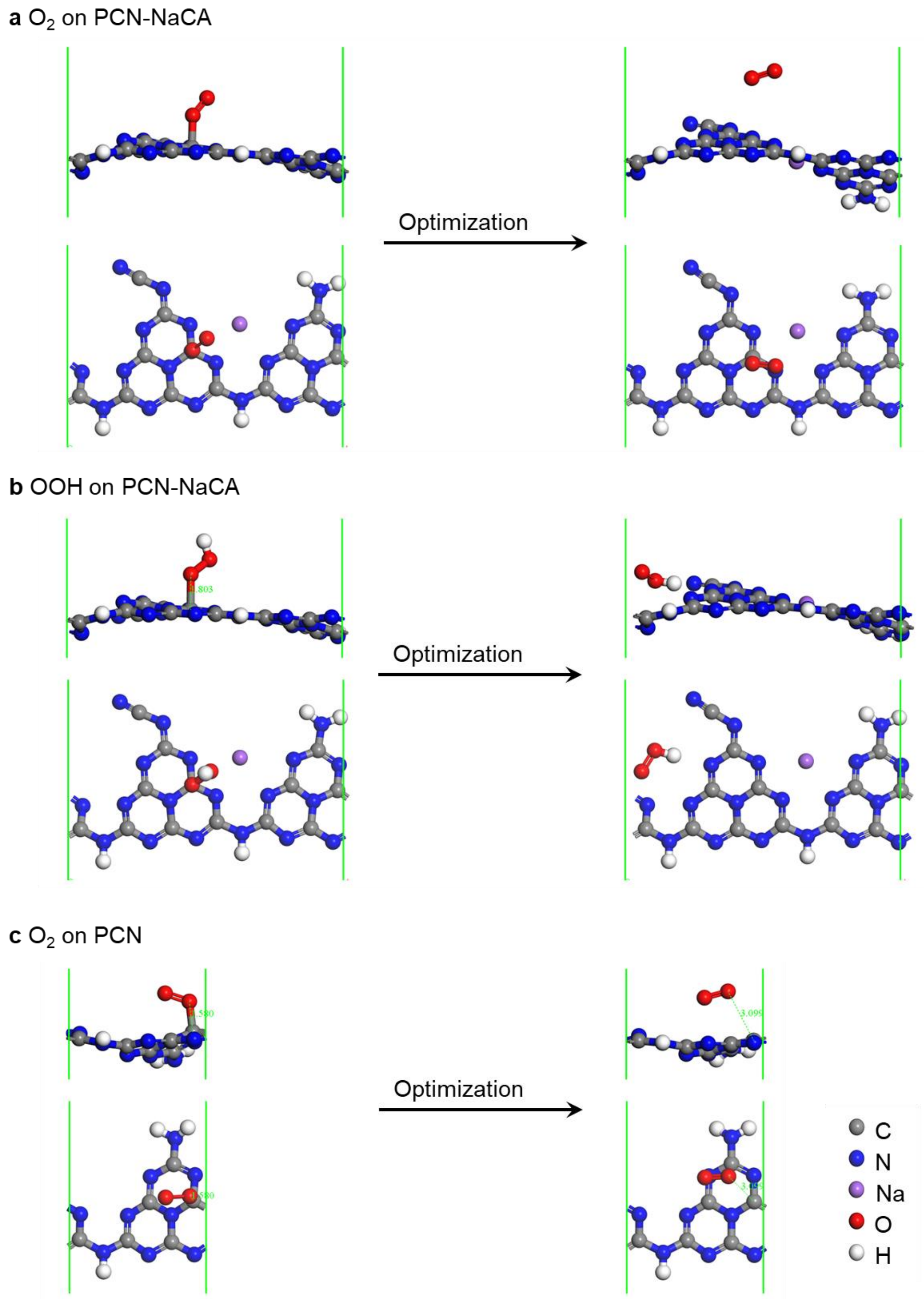

Figure S33. Initial structures and optimized configurations of O_2 on PCN-NaCA (**a**), OOH on PCN-NaCA (**b**), and O_2 on PCN (**c**) based on an electrically neutral cell.

Table S4. Adsorption energy values of O_2 and OOH on PCN-NaCA with different vacuum values. All results are in unit of eV.

Vacuum/Å	20	25	30
O_2	0.37	0.36	0.35
OOH	1.64	1.65	1.64

Notes: It was found that the variation of the adsorption energy is negligible, indicating that a vacuum with 2 nm is enough to eliminate the electrostatic interaction between the adjacent supercells.

Table S5. The adsorption energy (E_{ad}) of OOH on PCN-NaCA with different cell dimensions.

lattice /Å^3	13.99×23×20	13.99×30×25	13.99×35×30	13.99×40×35
E_{ad} /eV	1.64	1.64	1.63	1.63

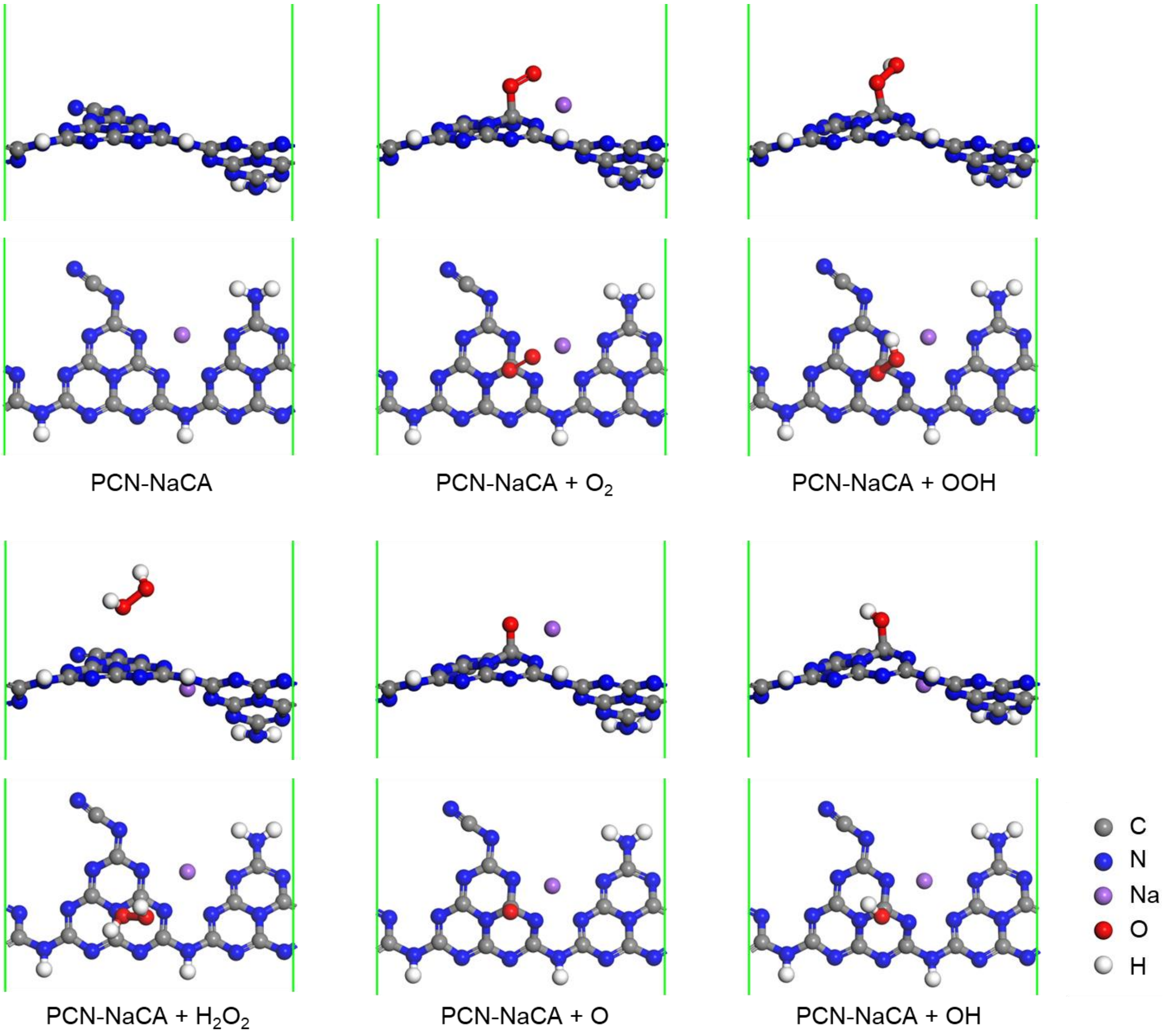

Figure S34. Optimized configurations of the intermediates on PCN-NaCA during ORR.

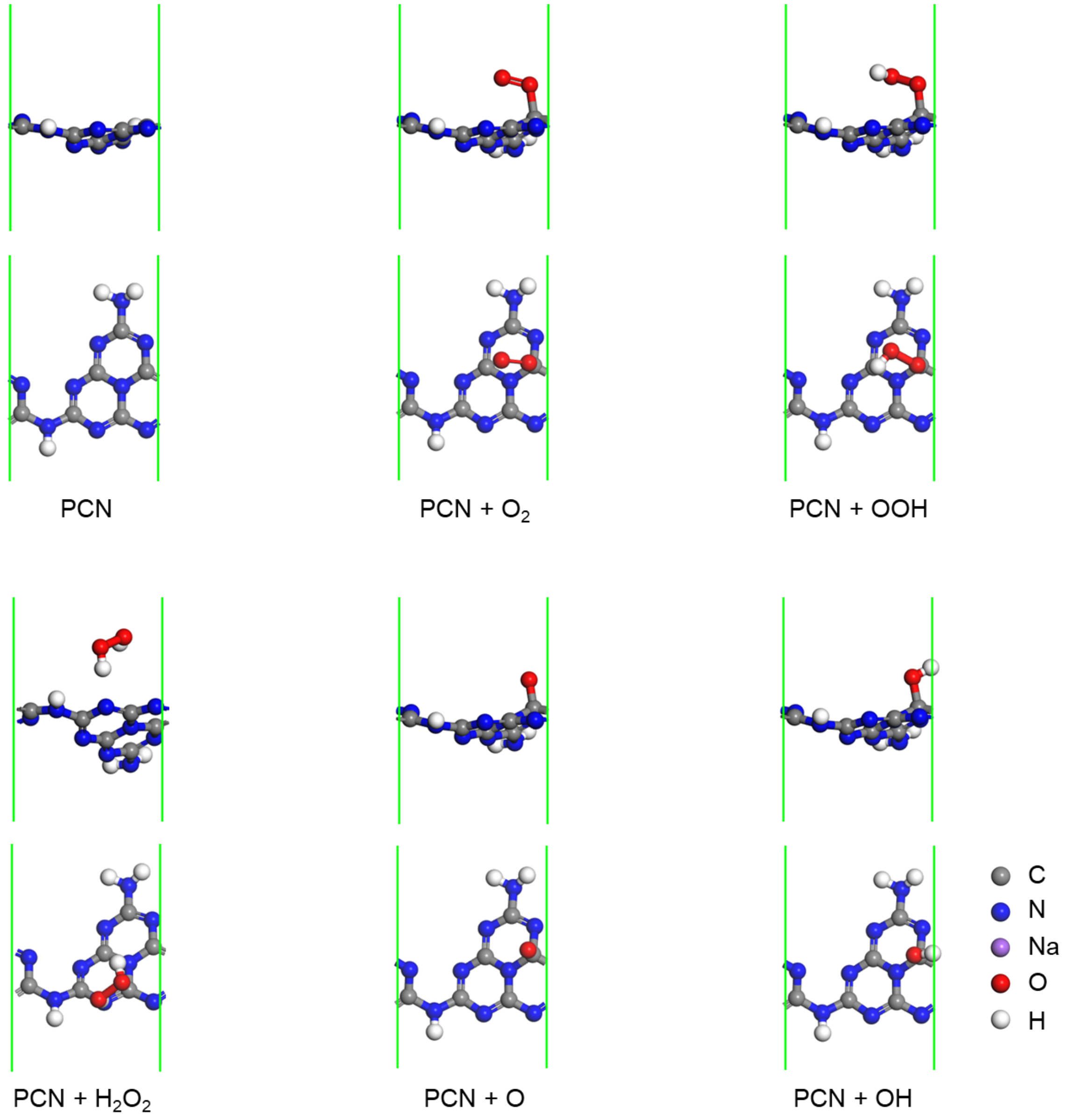

Figure S35. Optimized configurations of the intermediates on PCN during ORR.

Table S6. Mulliken charge population values of ORR intermediates on PCN and PCN-NaCA with an extra electron.

		O_2	OOH	O	OH
GGA-PBE	PCN	-0.443	-0.338	-0.536	-0.276
	PCN-NaCA	-0.519	-0.443	-0.617	-0.247
B3LYP	PCN	-0.477	-0.374	-0.413	-0.314
	PCN-NaCA	-0.479	-0.477	-0.708	-0.285

Notes: For double-checking the charge transfer results, hybrid functional B3LYP was employed as well. The charge transfer between ORR intermediates and substrate based on hybrid functional B3LYP is consistent with that based on GGA-PBE.

Table S7. Adsorption energy and interaction energy values of ORR intermediates on PCN and PCN-NaCA. All results are in unit of eV.

		O_2	OOH	O	OH
E_{ad}	PCN	1.54	2.35	4.48	3.03
	PCN-NaCA	0.37	1.64	3.76	2.48
E_{in}	PCN	2.41	3.19	5.28	3.79
	PCN-NaCA	2.97	3.01	5.44	3.75

References

1. Wang, X., Maeda, K., Chen, X., Takanabe, K., Domen, K., Hou, Y., Fu, X. and Antonietti, M. Polymer semiconductors for artificial photosynthesis: hydrogen evolution by mesoporous graphitic carbon nitride with visible light. *J. Am. Chem. Soc.* ***131***, 1680-1681 (2009).

2. Moon, G.-h., Kim, W., Bokare, A. D., Sung, N.-e. and Choi, W. Solar production of H_2O_2 on reduced graphene oxide–TiO_2 hybrid photocatalysts consisting of earth-abundant elements only. *Energy Environ. Sci.* ***7***, 4023-4028 (2014).

3. Wei, X., Xie, T., Peng, L., Fu, W., Chen, J., Gao, Q., Hong, G. and Wang, D. Effect of heterojunction on the behavior of photogenerated charges in Fe_3O_4@Fe_2O_3 nanoparticle photocatalysts. *J. Phys. Chem. C* ***115***, 8637-8642 (2011).

4. Jiang, T., Xie, T., Chen, L., Fu, Z. and Wang, D. Carrier concentration-dependent electron transfer in Cu_2O/ZnO nanorod arrays and their photocatalytic performance. *Nanoscale* ***5***, 2938-2944 (2013).

5. Delley, B. An all-electron numerical method for solving the local density functional for polyatomic molecules.

J. Chem. Phys. ***92***, 508-517 (1990).

6. Delley, B. From molecules to solids with the DMol^3 approach. *J. Chem. Phys.* ***113***, 7756-7764 (2000).

7. Perdew, J. P., Burke, K. & Ernzerhof, M. Generalized gradient approximation made simple. *Phys. Rev. Lett.* ***77***, 3865-3868 (1996).

8. Koelling, D. D. & Harmon, B. N. A technique for relativistic spin-polarised calculations. *J. Phys. C: Solid State Phys.* ***10***, 3107-3114 (1977).

9. Delley, B. The conductor-like screening model for polymers and surfaces. *Mol. Simul.* ***32***, 117-123 (2006).

10. Grimme, S. Semiempirical GGA-type density functional constructed with a long-range dispersion correction. *J. Comput. Chem.* **27**, 1787-1799 (2006).

11. Liu, W., Tkatchenko, A. & Scheffler, M. Modeling adsorption and reactions of organic molecules at metal surfaces. *Acc. Chem. Res.* ***47***, 3369-3377 (2014).

12. Liu, W., Filimonov, S. N., Carrasco, J. & Tkatchenko, A. Molecular switches from benzene derivatives adsorbed on metal surfaces. *Nat. Commun.* ***4***, 2569 (2013).

13. Nørskov, J. K. *et al.* Origin of the Overpotential for oxygen reduction at a fuel-cell cathode. *The Journal of Physical Chemistry B* ***108***, 17886-17892 (2004).

14. Rossmeisl, J., Nørskov, J. K., Taylor, C. D., Janik, M. J. & Neurock, M. Calculated phase diagrams for the electrochemical oxidation and reduction of water over Pt(111). *J. Phys. Chem. B* ***110***, 21833-21839 (2006).

15. Zheng, Y. *et al.* Nanoporous Graphitic-C_3N_4@carbon metal-free electrocatalysts for highly efficient oxygen reduction. *J. Am. Chem. Soc.* **133**, 20116-20119 (2011).

16 Chan, K. & Nørskov, J. K. Electrochemical barriers made simple. *J. Phys. Chem. Lett.* **6**, 2663-2668 (2015)

17. Yu, H., Shi, R., Zhao, Y., Bian, T., Zhao, Y., Zhou, C., Waterhouse, G. I. N., Wu, L.-Z., Tung, C.-H. and Zhang, T. Alkali-assisted synthesis of nitrogen deficient graphitic carbon nitride with tunable band structures for efficient visible-light-driven hydrogen evolution. *Adv. Mater.* ***29***, 1605148 (2017).

18. Gao, H., Yan, S., Wang, J., Huang, Y. A., Wang, P., Li, Z. and Zou, Z. Towards efficient solar hydrogen production by intercalated carbon nitride photocatalyst. *Phys. Chem. Chem. Phys.* ***15***, 18077-18084 (2013).

19. Guo, D. *et al.* Active sites of nitrogen-doped carbon materials for oxygen reduction reaction clarified using model catalysts. *Science* ***351***, 361-365 (2016).

20. Byrne, T. M. et al. Quaternary nitrogen activated carbons for removal of perchlorate with electrochemical regeneration. *Carbon* ***73***, 1-12 (2014).

21. Pels, J. R., Kapteijn, F., Moulijn, J. A., Zhu, Q. & Thomas, K. M. Evolution of nitrogen functionalities in carbonaceous materials during pyrolysis. *Carbon* ***33***, 1641-1653 (1995).

22. Yu, H., Shi, R., Zhao, Y., Bian, T., Zhao, Y., Zhou, C., Waterhouse, G. I. N., Wu, L.-Z., Tung, C.-H. and Zhang, T. Alkali-assisted synthesis of nitrogen deficient graphitic carbon nitride with tunable band structures for efficient visible-light-driven hydrogen evolution. *Adv. Mater.* ***29***, 1605148 (2017).

23. Sattler, A. & Schnick, W. On the formation and decomposition of the melonate ion in cyanate and thiocyanate melts and the crystal structure of potassium melonate, $K_3[C_6N_7(NCN)_3]$. *Eur. J. Inorg. Chem.* ***2009***, 4972-4981

(2009).

24 Zhang, P. et al. Heteroatom dopants promote two-electron O_2 reduction for photocatalytic production of h_2o_2 on polymeric carbon nitride. *Angew. Chem. Int. Ed*. **59**, 16209-16217 (2020).

25 Krivtsov, I. *et al.* Water-soluble polymeric carbon nitride colloidal nanoparticles for highly selective quasi-homogeneous photocatalysis. *Angew. Chem. Int. Ed.* **59**, 487-495 (2020).

26 Krishnaraj, C. *et al.* Strongly reducing (diarylamino)benzene-based covalent organic framework for metal-free visible light photocatalytic H_2O_2 generation. *J. Am. Chem. Soc.* **142**, 20107-20116 (2020).

27 Wu, Q. *et al.* A metal-free photocatalyst for highly efficient hydrogen peroxide photoproduction in real seawater. *Nat. Commun.* **12**, 483 (2021).

28 Shiraishi, Y. *et al.* Resorcinol–formaldehyde resins as metal-free semiconductor photocatalysts for solar-to-hydrogen peroxide energy conversion. *Nat. Mater.* **18**, 985-993 (2019).

29 Isaka, Y., Kawase, Y., Kuwahara, Y., Mori, K. & Yamashita, H. Two-phase system utilizing hydrophobic metal–organic frameworks (mofs) for photocatalytic synthesis of hydrogen peroxide. *Angew. Chem. Int. Ed.* **58**, 5402-5406 (2019).

30 Wei, Z. *et al.* Efficient visible-light-driven selective oxygen reduction to hydrogen peroxide by oxygen-enriched graphitic carbon nitride polymers. *Energy Environ. Sci.* **11**, 2581-2589 (2018).

31 Kofuji, Y. *et al.* Carbon nitride–aromatic diimide–graphene nanohybrids: metal-free photocatalysts for solar-to-hydrogen peroxide energy conversion with 0.2% efficiency. *J. Am. Chem. Soc.* **138**, 10019-10025 (2016).

32 Shiraishi, Y. *et al.* Sunlight-driven hydrogen peroxide production from water and molecular oxygen by metal-free photocatalysts. *Angew. Chem. Int. Ed.* **53**, 13454-13459 (2014).

33. Pettine, M., Campanella, L. & Millero, F. J. Reduction of hexavalent chromium by H_2O_2 in acidic solutions. *Environ. Sci. Technol.* ***36***, 901-907 (2002).

34. Kim, K. *et al.* Enhanced removal of hexavalent chromium in the presence of H_2O_2 in frozen aqueous solutions. *Environ. Sci. Technol.* ***49***, 10937-10944 (2015).

35. Zhang, G., Lin, L., Li, G., Zhang, Y., Savateev, A., Zafeiratos, S., Wang, X. and Antonietti, M. Ionothermal synthesis of triazine–heptazine-based copolymers with apparent quantum yields of 60 % at 420 nm for solar hydrogen production from “sea water”. *Angew. Chem. Int. Ed.* ***57,*** 9372-9376 (2018).

36. Lin, L., Yu, Z. and Wang, X. Crystalline carbon nitride semiconductors for photocatalytic water splitting. *Angew. Chem. Int. Ed.* ***58***, 6164-6175 (2019).

37. Kumar, P., Vahidzadeh, E., Thakur, U. K., Kar, P., Alam, K. M., Goswami, A., Mahdi, N., Cui, K., Bernard, G. M., Michaelis, V. K. and Shankar, K. C_3N_5: a low bandgap semiconductor containing an azo-linked carbon nitride framework for photocatalytic, photovoltaic and adsorbent applications. *J. Am. Chem. Soc.* ***141***, 5415-5436 (2019).
